# Genomes of coral dinoflagellate symbionts highlight evolutionary adaptations conducive to a symbiotic lifestyle

**DOI:** 10.1038/srep39734

**Published:** 2016-12-22

**Authors:** M. Aranda, Y. Li, Y. J. Liew, S. Baumgarten, O. Simakov, M. C. Wilson, J. Piel, H. Ashoor, S. Bougouffa, V. B. Bajic, T. Ryu, T. Ravasi, T. Bayer, G. Micklem, H. Kim, J. Bhak, T. C. LaJeunesse, C. R. Voolstra

**Affiliations:** 1 Red Sea Research Center, Division of Biological and Environmental Science and Engineering (BESE), King Abdullah University of Science and Technology (KAUST), Thuwal 23955-6900, Saudi Arabia; 2 Centre for Organismal Studies, Heidelberg University, 69120 Heidelberg, Germany; 3 Institute of Microbiology, Eidgenössische Technische Hochschule Zurich, Vladimir-Prelog-Weg 4, 8093 Zurich, Switzerland; 4 Computational Bioscience Research Center (CBRC), Computer, Electrical and Mathematical Sciences and Engineering Division (CEMSE), King Abdullah University of Science and Technology (KAUST), Thuwal 23955-6900, Saudi Arabia; 5 KAUST Environmental Epigenetics Program (KEEP), Division of Biological and Environmental Sciences and Engineering (BESE), King Abdullah University of Science and Technology (KAUST), Thuwal 23955-6900, Saudi Arabia; 6 GEOMAR Department: Evolutionary Ecology of Marine Fishes, GEOMAR Helmholtz Centre for Ocean Research, Kiel, Germany; 7 Department of Genetics, University of Cambridge, Cambridge CB2 3EH, UK; 8 Personal Genomics Institute, Genome Research Foundation, Suwon, Republic of Korea; 9 Department of Biology, The Pennsylvania State University, University Park, PA 16802, USA.

## Abstract

Despite half a century of research, the biology of dinoflagellates remains enigmatic: they defy many functional and genetic traits attributed to typical eukaryotic cells. Genomic approaches to study dinoflagellates are often stymied due to their large, multi-gigabase genomes. Members of the genus *Symbiodinium* are photosynthetic endosymbionts of stony corals that provide the foundation of coral reef ecosystems. Their smaller genome sizes provide an opportunity to interrogate evolution and functionality of dinoflagellate genomes and endosymbiosis. We sequenced the genome of the ancestral *Symbiodinium microadriaticum* and compared it to the genomes of the more derived *Symbiodinium minutum* and *Symbiodinium kawagutii* and eukaryote model systems as well as transcriptomes from other dinoflagellates. Comparative analyses of genome and transcriptome protein sets show that all dinoflagellates, not only *Symbiodinium*, possess significantly more transmembrane transporters involved in the exchange of amino acids, lipids, and glycerol than other eukaryotes. Importantly, we find that only *Symbiodinium* harbor an extensive transporter repertoire associated with the provisioning of carbon and nitrogen. Analyses of these transporters show species-specific expansions, which provides a genomic basis to explain differential compatibilities to an array of hosts and environments, and highlights the putative importance of gene duplications as an evolutionary mechanism in dinoflagellates and *Symbiodinium*.

Dinoflagellates are ubiquitous freshwater and marine protists, having great economic and ecological importance^1^. Over half a century of extensive research into their biology shows that they defy many cellular and genetic traits commonly attributed to eukaryotic cell biology and function^2^,^3^. The organization and regulation of genes in particular is different from most other eukaryotes, and include non-canonical intron splicing, the existence of unusual upstream promoter regions for many genes, DNA that contains 5-hydroxymethyluracil (replacing 12–70% of thymidine), and a greater reliance on translational – rather than transcriptional – gene regulation^2^. Moreover, dinoflagellate genomes have among the highest levels of DNA acquired through horizontal gene transfer (HGT)^4^. The increased study of dinoflagellate genomics will enhance basic understanding of the evolution and functionality of eukaryotic genomes and may help to further our knowledge about dinoflagellate physiology and ecology.

Indeed, there are a growing number of transcriptomes and expression data available for many dinoflagellates^5^,^6^. However, these analyses lack the scaffolding of a reference genome to know how many genes exist, relate how genes are organized and positioned relative to each other, and allow the future application of transgenic approaches for the interpretation of gene function^7^. The major obstacle that limits the analysis of dinoflagellate genome organization and function is that their genomes are unusually large (~1.5–250 Gb) relative to other eukaryotes^8^. Until the sequencing of large and highly repetitive genomes is made easier, comparative genomics among dinoflagellates will be limited to those species with the smallest genomes. The genomes of species in the genus *Symbiodinium* are among the smallest (~1–5 Gb) relative to other dinoflagellates, which corresponds to their small cell size^9^. For this reason, the first available draft genome of a dinoflagellate was that of *Symbiodinium minutum*^10^, and soon followed by *Symbiodinium kawagutii*^11^.

Members of the genus *Symbiodinium* occur often in widespread symbioses with metazoans in the phylum Cnidaria as well as with many other animals and protists^12^. Their symbioses with reef-building corals create the foundation for one of the most diverse and productive marine ecosystems on the planet – coral reefs. Growing concerns over climate change and reef degradation heighten the need to understand the genomic underpinning of physiological differences among the vast number of *Symbiodinium* species. The large numbers of available cultures representing numerous closely and distantly related species and strains constitute a critical resource and model system for comparative genomics among dinoflagellates^13^. The draft genomes of *S. minutum* and *Symbiodinium kawagutii* confirmed that the genomic makeup of *Symbiodinium* is similar to other dinoflagellates, including the presence of spliced leader sequences and non-canonical splice sites, and a prevalence of genes acquired from bacteria^10^,^11^. In addition, large contigs from the genome of *S. minutum* indicated a strong tendency for unidirectionally aligned genes.

The publication of the genomes of *S. minutum* and *S. kawagutii* has been accompanied in recent years by a number of studies that have analyzed and compared the transcriptomes among distantly related species^14^,^15^,^16^,^17^,^18^,^19^. Their long evolutionary divergence was reflected in the considerable differences found between their transcriptome profiles^14^,^18^. However, the limited availability of genomes prevented making further generalities about the organization and function of *Symbiodinium* genomes, how this translates into their ability to form environmentally stable symbioses with specific hosts, and whether gene content and the representation of biochemical pathways is a common feature of all *Symbiodinium*, or even dinoflagellates in general.

To further advance our capacity for comparative genomics and relating transcriptional profiling with genome wide analyses, we sequenced the genome of *Symbiodinium microadriaticum*^20^. This species occurs in symbioses with the jellyfish *Cassiopea xamachana*^21^. *S. microadriaticum* is a member of the most ancestral lineage, Clade A, while *S. minutum* is a representative member of Clade B^22^ and *S. kawagutii* of the more derived Clade F^11^; these lineages shared a common ancestor at least 45–55 MYA^23^. Accordingly, comparing the genomes of *S. microadriaticum*, *S. minutum*, and *S. kawagutii* provides an opportunity to determine whether gene organization and content is conserved across lineages separated by tens of millions of years. Moreover, it allows for the comparison of their corresponding gene sets to transcriptomes from other dinoflagellates to unequivocally assess which features are shared among dinoflagellates and which are specific to *Symbiodinium*, potentially revealing distinct characteristics that contribute to their ecological success as intracellular symbionts.

## Results

### Genome size of *S. microadriaticum*

The draft genome of *S. microadriaticum* (strain CCMP2467) encompasses 808 Mbp of the 1,100 Mbp genome (based on *k*-mer distribution), of which 746 Mbp were assembled into contigs (Supplemental Information, Table S1). A subsequent FACS (Fluorescence-activated cell sorting) analysis estimated the genome size at 1,400 Mbp, indicating that either *k*-mer based estimates in *Symbiodinium* might underestimate dinoflagellate genome sizes or that FACS based analyses include extra-nuclear DNA (Supplemental Information, Fig. S1). The scaffold N50 of the assembled genome is 573.5 kbp featuring a contig N50 of 34.9 kbp and encoding for 49,109 genes, of which 24,610 (~50%) show homology to genes from available databases (Table 1, Supplemental Information, Table S1 and Table S2). This compares well with the ~609 Mbp draft genome containing 41,925 genes (contig N50 of 62.7 kbp and scaffold N50 of 125.2 kbp) of *S. minutum* and the ~935 Mbp genome containing 36,850 genes (contig N50 of 47.1 kbp and scaffold N50 of 380.9 kbp) of *Symbiodinium kawagutii*. Notably, GC content was considerably higher in *S. microadriaticum* (50.5%) than in *S. minutum* (43.5%) and *S. kawagutii* (45.5%).

### Genome organization of *S. microadriaticum*

To estimate the completeness of the assembled genome, we analyzed the presence of 458 highly conserved eukaryotic genes^24^. This analysis was performed on the genomes of *S. microadriaticum*, *S. minutum*, and *S. kawagutii* to ensure similar completeness for all subsequent comparative analyses. We identified 437 (95.4%), 434 (94.8%), and 383 (83.6%) homologs for *S. microadriaticum*, *S. minutum*, and *S. kawagutii* respectively, of which 373 (81.4%) were common between all three species (Dataset S1.1). A strong directionality in gene orientation was observed for *S. microadriaticum* (featuring an average of 2.32 gene orientation changes per 10-gene window), but was significantly less pronounced (*χ*^*2*^ test, *p*-value < 2.2 × 10^−16^) than in *S. minutum* (0.64 changes), although similar to *S. kawagutii* (2.11 changes) (Supplemental Information, Fig. S2). Since the *Symbiodinium* species belong to clades that are evolutionarily distant from each other (45–55 MYA)^23^, we wanted to assess whether gene order was a conserved feature between the three species. Syntenic blocks of at least five genes with similarities <1e^−5^ were identified from all three genomes using MCScanX^25^. These analyses revealed startlingly few and short synteny blocks between *S. microadriaticum* and *S. minutum* (349 blocks ≤10 genes), and even fewer regions could be identified in any of the comparisons to *S. kawagutii* (*S. microadriaticum* vs. *S. kawagutii*: 166 blocks ≤10 genes; *S. minutum* vs. *S. kawagutii*: 222 blocks ≤10 genes) (Supplemental Information, Table S3, Fig. S3). This lack of syntenic conservation indicates substantial genomic differences between these species. Comparison of the gene densities between the three genomes showed pronounced differences with 61 vs. 68 vs. 39 genes per Mb for *S. microadriaticum, S. minutum*, and *S. kawagutii*, respectively. Patterns of canonical and non-canonical splice sites were highly similar between all three species (Supplemental Information, Fig. S4) with the exception of *S. kawagutii*, which contained some donor sites starting with cytosine instead of the canonical guanine.

### Genic composition of *Symbiodinium* genomes

**Figure 1:**
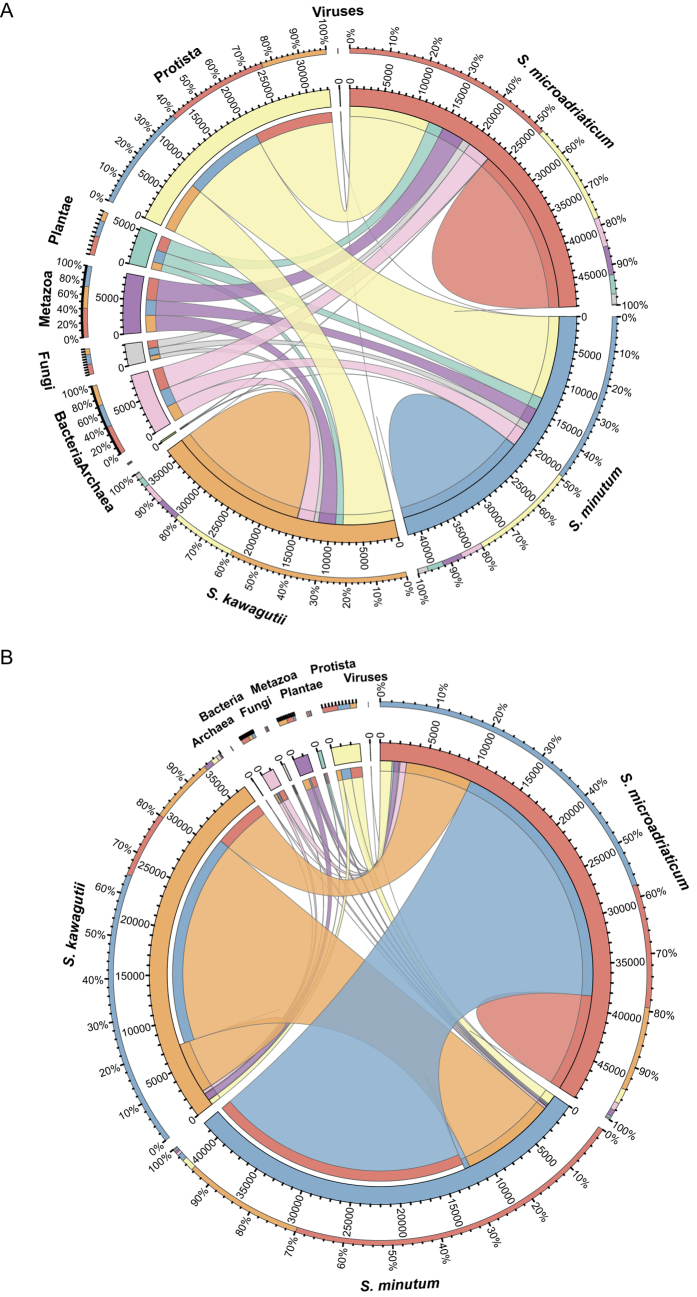
Comparison of genomic composition of the genomes of *Symbiodinium microadriaticum, Symbiodinium minutum*, and *Symbiodinium kawagutii.* Genes were classified by best hits against nr database into seven kingdom/subkingdom-level organismal groups: Archaea, Bacteria, Fungi, Metazoa, Plantae, Protista, and Viruses. Chord diagrams show (**A**) the fairly even number of matches from both *Symbiodinium* species to these seven groups respectively, and (**B**) the large overlap between *Symbiodinium* species when they were allowed to match against each other.

To further understand genic composition of *Symbiodinium* genomes, we performed a BLASTP analysis using the gene sets encoded in all three genomes. The resulting best hits were categorized and grouped by their putative phylogenetic origin into 7 groups (i.e., Viruses, Bacteria, Archaea, Protista, Viridiplantae, Metazoa, and Fungi) based on their taxonomically best match (Fig. 1A). In addition, and to examine the similarity of all three genomes, we performed the same analysis, but included hits between the *Symbiodinium* genomes to highlight commonalities and differences in the genic content (Fig. 1B). All genomes showed a highly similar taxonomic distribution of their respective gene sets (Fig. 1A). As expected, the vast majority of the genes matched known proteins from other protists (~25% of all gene models in *S. microadriaticum*, ~30% in *S. minutum*, and ~21% in *S. kawagutii*), followed by bacteria (8% vs. 7% vs. 5%, respectively). All species had a similar amount of genes corresponding to metazoan genes (7% vs. 6% vs. 7%, respectively), while genes with similarities to fungi, archaea, and viruses were generally few and accounted for less than 3%. The inclusion of matches to the other *Symbiodinium* genomes emphasized the relatedness of the three species (Fig. 1B). Approximately 58% of the *S. microadriaticum* genes had their best hit in *S. minutum*, while ~15% had their best match to *S. kawagutii* genes. Reciprocally, ~70% of the *S. minutum* genes had their best hit in *S. microadriaticum*, but only ~24% had a better hit to *S. kawagutii*, although *S. microadriaticum* is ancestral to both other species. Interestingly, we found that while the majority of *S. minutum* genes had their best hit to genes from *S. microadriaticum*, most of the *S. kawagutii* genes had their best match to genes from *S. minutum* (~64%) and considerably less genes hat better hits to *S. microadriaticum* (~16%). Further, although the majority of genes from all three species had matches at least in one of the other *Symbiodinium* species, we still found a considerable amount of genes in *S. microadriaticum* (21%) and *S. kawagutii* (15%), but not in *S. minutum* (2%), without significant hits at all, suggesting that these genes might be putatively species- or lineage-specific.

### Functional gene content of *Symbiodinium* relative to other eukaryotes

**Figure 2:**
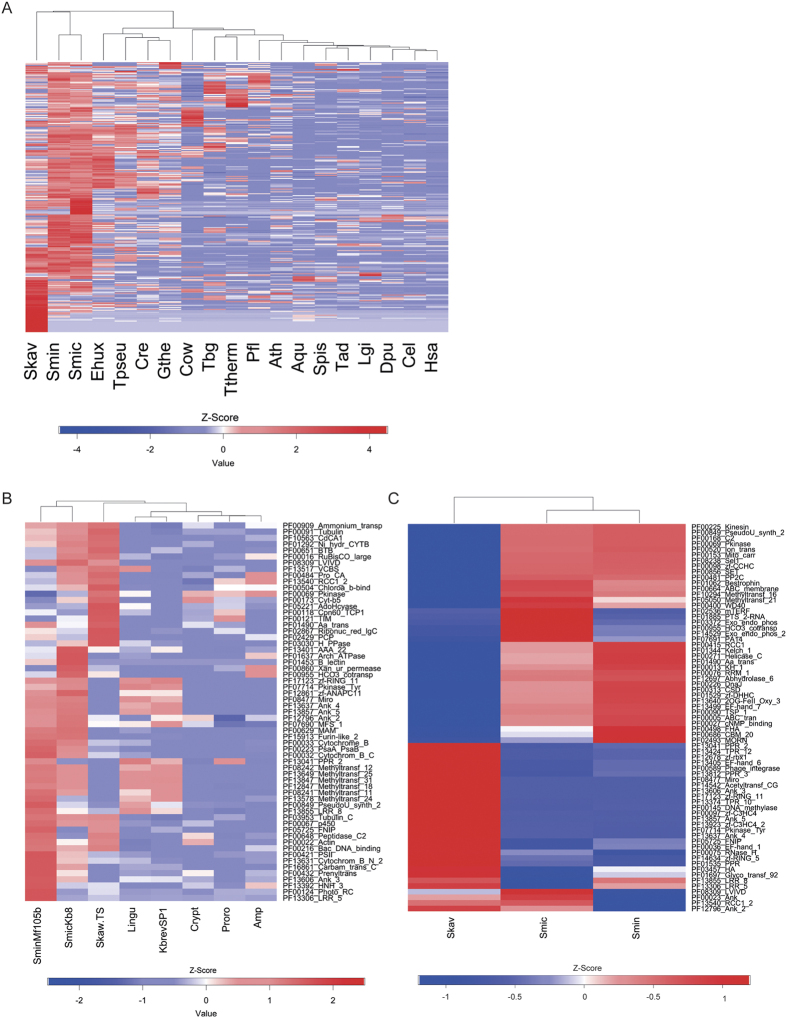
Pfam domain enrichment in protein sets of *Symbiodinium microadriaticum, Symbiodinium minutum*, and *Symbiodinium kawagutii* (**A**) in comparison to 16 reference eukaryotic genomes, (**B**) in comparison to other dinoflagellate transcriptomes, (**C**) among *Symbiodinium* genomes. Rows indicate distinct Pfam domains. Domain enrichment was estimated via Fisher’s exact test on domain ratios, colors represent z-scores, and samples were clustered by Euclidean distance. Species names providing reference genomes are abbreviated as per the following: *Symbiodinium kawagutii* (Skav), *Symbiodinium minutum* (Smin), *Symbiodinium microadriaticum* (Smic), *Emiliania huxleyi* (Ehux), *Thalassiosira pseudonana* (Tpseu), *Chlamydomonas reinhardtii* (Cre), *Guillardia theta* (Gthe), *Capsaspora owczarzaki* (Cow), *Trypanosoma brucei gambiense* (Tgb), *Tetrahymena thermophila* (Ttherm), *Plasmodium falciparum* (Pfl), *Arabidopsis thaliana* (Ath), *Amphimedon queenslandica* (Aqu), *Stylophora pistillata* (Spis), *Trichoplax adhaerens* (Tad), *Lottia gigantea* (Lgi), *Daphnia pulex* (Dpu), *Caenorhabditis elegans* (Cel), *Homo sapiens* (Hsa). Species names providing reference transcriptomes are abbreviated as per the following: *Symbiodinium microadriaticum* strain Kb8 (SmicKb8), *Symbiodinium minutum* strain Mf1.05b (SminMf1.05b), *Symbiodinium kawagutii* (Skaw.TS), *Karenia brevis* strain SP1 (KbrevSP1), *Lingulodinium polyedrum* (Lingu), *Prorocentrum minimum* (Proro), *Amphidinium carterae* (Amp), and *Crypthecodinium cohnii* (Crypt). A complete list of pfam domains and pfam annotations for all analyses are accessible in the supplement (Dataset S1.2, Dataset S1.3, Dataset S.14, Supplemental Information, Table S5).

In order to identify specific molecular functions that are enriched in *Symbiodinium*, we compared the relative frequencies of protein domains obtained from the translated genomic gene sets of *S. microadriaticum*, *S. minutum*, and *S. kawagutii* to reference genomic protein sets from 16 organisms (Fig. 2A, Supplemental Information, Table S4, Dataset S1.2). Our analysis produced 280 significantly enriched protein domains (FDR < 0.001) and emphasized just how distinct *Symbiodinium* genomes are, even relative to other protist genomes (Fig. 2A, Supplemental Information, Table S5). In line with previous findings^11^, we found a surprisingly large amount of domains involved in transmembrane transport to be highly enriched in *Symbiodinium* (20 out of 280). These included transporter domains specific for bicarbonate, ammonium, phosphate, lipids, glycerol, amino acids, choline, sugars, and sulfates (among others), as well as the more general ABC and ion transporters (Supplemental Information, Table S5). We also identified enrichment of carbonic anhydrases in the genomes of *S. microadriaticum, S. minutum*, and *S. kawagutii* in comparison to other eukaryotic genomes (Supplemental Information, Table S5).

### Functional gene content of *Symbiodinium* relative to other dinoflagellates

Due to the absence of other dinoflagellate genomes, we could not rule out whether the observed enrichment of transmembrane transport and other domains is specific to the genus *Symbiodinium* or a general trait of dinoflagellates (see above). Therefore, we conducted an enrichment analysis using transcriptomic data of the dinoflagellates *Karenia brevis*^26^, *Lingulodinium polyedrum*^27^, *Amphidinium carterae*^28^, *Crypthecodinium cohnii*^28^, and *Prorocentrum minimum*^28^, and compared these to available transcriptomes from *S. microadriaticum* (strain KB8)^14^, *S. minutum* (strain Mf1.05b)^14^, and *S. kawagutii*^11^ (Supplemental Information, Supplemental Analysis, Fig. S5, Dataset S1.3). This analysis identified 61 protein domains (FDR < 0.001) enriched in at least one of the *Symbiodinium* species (Fig. 2B, Supplemental Information, Table S5), of which 40 were shared with the previous comparison to (non-dinoflagellate) eukaryotes. We designated these 40 domains as *Symbiodinium*-specific enriched domains (Supplemental Information, Table S5). The enriched domains confirmed that bicarbonate transporters (PF00955 HCO3_cotransp), carbonic anhydrases (PF00484 Pro_CA), and ammonium transporters (PF00909 Ammonium_transp) are specifically enriched in *Symbiodinium* when compared to other eukaryotes, including dinoflagellates. We could also confirm that Ankyrin domains (Ank), regulator of chromatin condensation (RCC1) repeat domains, and Methyltransferase domains are specifically enriched in *Symbiodinium*^10^,^11^,^16^,^29^, among others (Supplemental Information, Table S5). Importantly, the 40 domains did not contain any of the transmembrane transporters (i.e., lipid, glycerol, phosphate, ion, sulfate, ABC transporters, etc.) that were previously reported to be specifically enriched in *Symbiodinium*^11^ with the exception of an amino acid transporter domain (PF01490 Aa_trans) that was highly prevalent in *S. minutum* and *S. kawagutii,* but not *S. microadriaticum*. Further, we did not find enrichment of protein domains associated with oxidative stress in *Symbiodinium* (e.g., HSP70, HSP90, DnaJ, SOD, APx, catalase, thioredoxin, etc.)^14^,^16^,^29^,^30^,^31^,^32^, suggesting that these domains are a common feature for many dinoflagellates^11^.

### Functional gene content differences between *Symbiodinium* species

To increase our understanding regarding *Symbiodinium* species differences, we compared relative domain abundances between *S*. *microadriaticum, S. minutum*, and *S. kawagutii*. This analysis revealed 81 protein domains (FDR < 0.01) with significant differences across the three species (Fig. 2C, Dataset S1.4). Interestingly, we found several transporter domains to be significantly differentially abundant in the three species including bicarbonate and amino acid transporters as well as the more general ABC and ion transporters. Although we did not find specific enrichment of stress related protein domains between *Symbiodinium* and other dinoflagellates (see above), the number of the stress-associated chaperone domain DnaJ (PF00226 DnaJ) differed significantly between *Symbiodinium* species (Fig. 2C, Dataset S1.4).

### *Symbiodinium*-specific differences in genes involved in carbon acquisition

**Figure 3:**
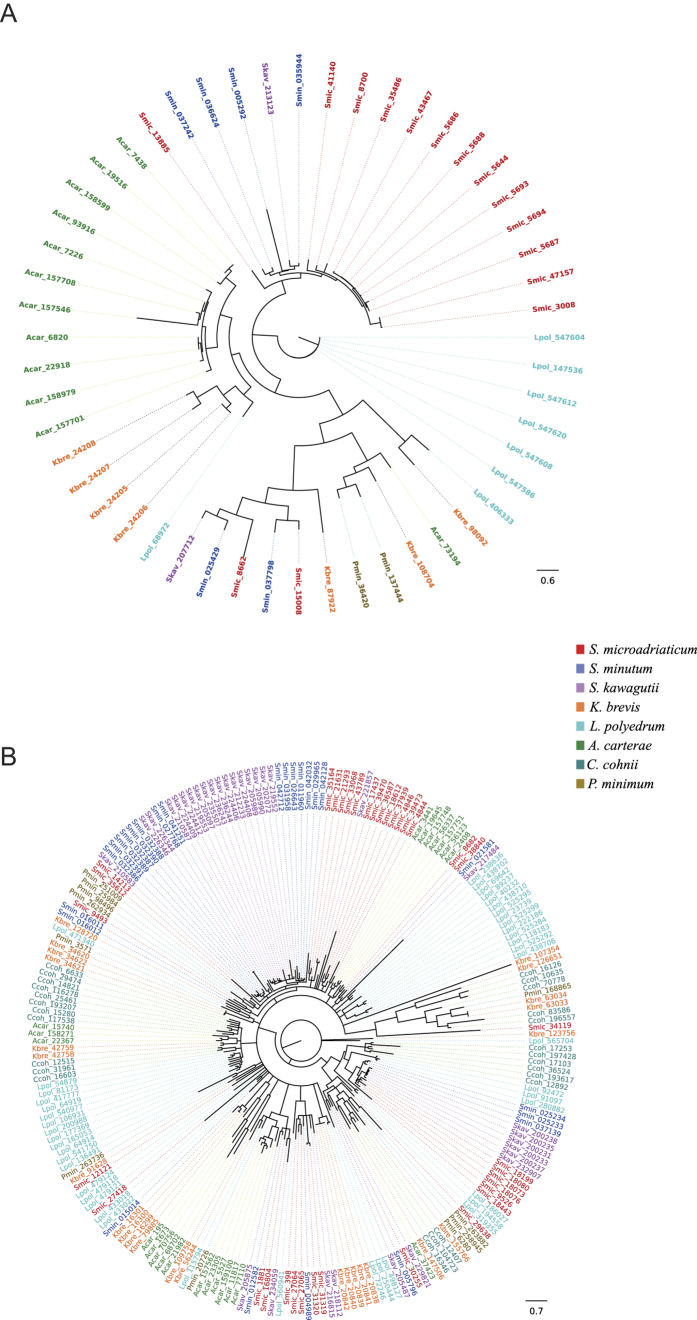
Maximum-likelihood trees (1,000 bootstraps) for (**A**) bicarbonate transporters (PF00955 HCO3_cotransp) and (**B**) ammonium transporters (PF00909 Ammonium_transp), present in the genomes of *Symbiodinium microadriaticum*, *Symbiodinium minutum*, *Symbiodinium kawagutii* and the transcriptomes of *Karenia brevis, Lingulodinium polyedrum*, *Amphidinium carterae*, *Crypthecodinium cohnii*, and *Prorocentrum minimum*. Phylogenetic grouping of bicarbonate and ammonium transporters by species indicates lineage specific duplications in all dinoflagellate species. Only genes and transcripts with transporter domains with e-values > 10^−15^ and lengths above 150 amino acids were selected for the analysis. Species-specific gene duplications are colored according to species and edges with bootstrap support below 50 are collapsed. Files for phylogenetic tress are provided in the supplement (Supplemental Information, File S1 and File S2).

The functional importance of nutrient exchange for the host-algal symbiosis has been broadly shown^11^,^33^,^34^,^35^. In our enrichment analyses, bicarbonate transporters and carbonic anhydrases were among the *Symbiodinium*-specific enriched domains (Supplemental Information, Table S5). The proteins containing these domains form integral parts of carbon-concentrating mechanisms (CCMs) that play an important role in the acquisition of inorganic carbon for photosynthesis in many algae^36^,^37^,^38^,^39^. We found bicarbonate transporter domains (PF00955 HCO3 cotransp) to be highly enriched in *Symbiodinium microadriaticum*, but significantly less so in *S. minutum* and *S. kawagutii* (52 vs. 15 vs. 4, Dataset S.1.2), indicating pronounced differences between the three *Symbiodinium* species (Dataset S1.4). To further understand species differences, we conducted a phylogenetic analysis of bicarbonate transporters (Fig. 3A, Dataset 1.5, Supplemental Information, File S1). This analysis showed that genes from *S. microadriaticum, S. minutum*, and *S. kawagutii* generally clustered together and apart from other dinoflagellate sequences, supporting the phylogenetic relatedness of these species. Interestingly, we observed that bicarbonate transporter genes tended to cluster by species, implicating that several of these genes arose through clade- or species-specific gene duplications (Fig. 3A). We further corroborated this result by phylogenetic analysis of bicarbonate transporters from species in *Symbiodinium* from Clades A, B, C, and D using transcriptome data from^16^, where we found that transcripts also tended to cluster by species (Supplemental Information, Fig. S6A). Interestingly, analysis of transcriptomic data derived from *Symbiodinium microadriaticum* (Clade A1) subjected to nine different experimental treatments (4 °C 4hs, 16 °C 4hs, 34 °C 12hs, 36 °C 4hs, 20 g/L NaCl 4hs, 60g/L NaCl 4hs, dark cycle, dark stress, light cycle)^17^ showed that the bicarbonate transporters encoded by two genomic genes (Smic15008 and Smic8700) were differentially expressed in response to coral bleaching relevant stressors cold (4 °C) and heat stress (34 °C and 36 °C). In line with the enrichment of bicarbonate transporters, we also found carbonic anhydrase domains (PF00484 Pro_CA) to be overrepresented in all three *Symbiodinium* species. In contrast to the bicarbonate transporters, however, we found no significant differences in the relative representation of these domains in any of the three *Symbiodinium* species (Fig. 2C).

### *Symbiodinium*-specific differences in genes involved in nitrogen acquisition

Nitrogen is a limiting factor for growth in the oligotrophic environment of tropical seas. Accordingly, many marine organisms acquired the ability to assimilate nitrogen from inorganic sources^40^,^41^. In line with that we found ammonium transporters (PF00909 Ammonium_transp) among the *Symbiodinium*-specific enriched domains (Supplemental Information, Table S5) and highly enriched in all *Symbiodinium* species. We compared the amount of ammonium transporter domains between *Symbiodinium* and the genomes of three symbiotic cnidarians, i.e. the scleractinian corals *Stylophora pistillata*^42^,^43^ and *Acropora digitifera*^44^ as well as the symbiotic anemone *Aiptasia*^45^ (Dataset S1.2). While the cnidarian genomes appear to encode for 18, 7, and 15 ammonium transporter domains respectively, we identified 68, 42, and 46 such domains in the genomes of *S. microadriaticum*, *S. minutum,* and *S. kawagutii*. Phylogenetic analysis of these transporters provided a similar result as for the bicarbonate transporters showing strong clustering of sequences by *Symbiodinium* species, indicating substantial intra-cladal or even lineage- or species-specific duplications (Fig. 3B, Dataset S1.6, Supplemental Information, File S2). As with the analysis of bicarbonate transporters, *Symbiodinium*-specific differences in genes involved in nitrogen acquisition could be corroborated by phylogenetic analysis of bicarbonate transporters of species across clades A, B, C, and D using transcriptome data from ref. 16, where it was found that genes clustered by species indicating species-/clade-specific duplication (Supplemental Information, Fig. S6B). Similar to the expression analysis of bicarbonate transporters, we found several ammonium transporters (Smic33068, Smic43789, Smic8682, and Smic37939) to be differentially expressed in response to heat stress (36 °C), but not in any of the other experimental treatments^17^.

## Discussion

A genomic understanding of dinoflagellates has long been elusive due to their large genomes and unusual genome structure^10^. Our comparative genome and transcriptome analyses incorporating *Symbiodinium* and dinoflagellate sequence data allowed us to differentiate *Symbiodinium*-specific and general dinoflagellate-specific traits in order to further understand and pinpoint features that explain the success of dinoflagellates in general and the endosymbiosis lifestyle of *Symbiodinium* in particular. Of equal importance, we identified pronounced genomic differences between *Symbiodinium* species that may hold implications for knowledge on their different physiologies and ecologies.

### Genome organization

The analysis of the previously published *S. minutum* and *S. kawagutii* genomes highlighted several genomic particularities representing presumably *Symbiodinium*-specific traits. Our comparative analyses confirmed that this is the case for the high abundance of non-canonical splice sites, but it does not necessarily apply for the exceptionally strong tendency towards unidirectionality of gene arrangements in *S. minutum* for instance. The difference in gene orientation frequency is in line with our overall finding of high genomic dissimilarity, despite a seemingly considerable genic similarity. Although neither of the genome assemblies encompass the entire estimated genome sizes, they showed similar completeness based on the CEG analysis. However, the genome composition analysis highlighted that substantially more *S. minutum* genes had their best match to the phylogenetically more distant *S. microadriaticum*, rather than *S. kawagutii* (70% vs. 24%), while most *S. kawagutii* genes had their best match to *S. minutum* (64%). The stark discrepancy of the overall number of gene matches between the *S. minutum* and *S. kawagutii* gene sets might indicate accelerated evolution in the *S. kawagutii* lineage. This is further supported by the observed differences in synteny conservation between the three species representing three clades (Supplemental Information, Table S3) that show that *S. minutum* is more similar to *S. microadriaticum* than to *S. kawagutii*. However, we cannot fully exclude that other factors such as the lower number of genes found in *S. kawagutii* or differences in genome assembly and gene prediction methods might have also contributed to the patterns observed here.

### Enrichment of domains and transporters conducive to an endosymbiotic lifestyle

Our enrichment analysis identified several common domains as well as important differences between *Symbiodinium* and other dinoflagellates. Arguably, the comparison between *Symbiodinium* and other dinoflagellates is important if we are to understand which traits potentially represent adaptations conducive to an endosymbiotic lifestyle by means of enriched protein domain analyses. Unfortunately, this analysis is currently limited to the use of transcriptomic data for comparative analyses between dinoflagellates due to the lack of non-*Symbiodinium* dinoflagellate genomes. It should be noted that using transcriptomic data is not free of caveats and can only provide an approximation, especially considering the high gene duplication rates that potentially impair the assembly of transcripts originating from recently duplicated genes. Resolving such transcripts is likely further affected by our clustering approach, which might combine transcripts originating from different genes into the same locus, thus underestimating the true number of gene copies present in the genome. However, by comparing transcriptomes from sequenced *Symbiodinium* genomes to other dinoflagellate transcriptomes, we tried to minimize this bias, at least to the extent that we can assess the margin of error. This is highlighted in the correlation analyses of the *Symbiodinium* genomes and respective transcriptomes, which showed strong and significant, but far from perfect correlations (Supplemental Information, Fig. S5).

In line with previous reports^11^, we found an unexpected prevalence of transmembrane transporters involved in the translocation of diverse nutrients and ions in *Symbiodinium*. However, fine-scale comparative analysis with multiple dinoflagellate species revealed that this feature seems to be shared across all dinoflagellates, and hence, does not represent a specific adaptation of *Symbiodinium* to an endosymbiotic lifestyle as previously suggested^11^. Accordingly, our findings argue that dinoflagellates in general harbor genomic traits that are conducive to the evolution of symbiotic lifestyles (reviewed in ref. 46). At the same time, our enrichment analysis revealed a surprisingly rich repertoire of *Symbiodinium*-specific transporter expansions, i.e. bicarbonate- and ammonium-related domains. These transporters are likely to play a fundamental role in symbiotic relationships as they represent key elements for the production of photosynthates, their exchange, and organismal growth.

Inorganic carbon is a key limiting factor of photosynthesis^47^ and its constant provision in the hyperoxic environment of illuminated coral tissues is highly important for photosynthesis and protection from photodamage^48^,^49^. It is therefore not unexpected to find bicarbonate transporters and carbonic anhydrases to be enriched in *Symbiodinium*. Interestingly though, we found significant differences in the amount of bicarbonate transporters between the three *Symbiodinium* species analyzed. The phylogenetic analysis of these transporters further suggests that many of these genes arose through independent gene duplications in the respective species (lineages), rather than gene loss.

Significant differences in the capacities of coral host and *Symbiodinium* to assimilate ammonium were shown in a study by ref. 40. The authors suggested different scenarios as an explanation including the possibility of higher ammonium transporter activity in the symbiont. Our results strongly suggest that the observed differences in ammonium uptake could be caused by the significantly higher number of ammonium transporters present in *Symbiodinium*. We hypothesize that the increased ability of *Symbiodinium* in transporting ammonium allows the symbiont to act as an “ammonium sink” within coral host cells, thereby increasing the nitrogen efficiency of the holobiont by creating a strong ammonium gradient that facilitates diffusion of ammonium from the seawater into the host tissue as previously proposed^50^. It is tempting to assume that this could have evolved as a way to counteract nitrogen limitation by the host (“selfish symbiont”), while ultimately becoming central to the nitrogen efficiency of the holobiont and its increased ability to acquire nitrogen from the environment. Indeed, it has been observed that increases in available ammonium lead to surges in *Symbiodinium* cell densities within the host tissue^51^,^52^,^53^. Interestingly, it has also been proposed that such nitrogen-induced increases in *Symbiodinium* cell densities might lead to potential carbon and phosphate limitation due to the increased demand of the growing symbiont population. This, in turn, has been speculated to result in photoinhibition and consequently bleaching of the coral host^49^,^53^,^54^.

What follows is that the observed differences in the number of encoded bicarbonate, ammonium, and other domains might provide a genomic basis to explain species-specific physiological traits that affect symbiosis and host range of different *Symbiodinium*. Furthermore, these traits might also contribute to the susceptibility of certain host-symbiont combinations to environmental stressors such as temperature and eutrophication.

### Pervasive gene duplication in dinoflagellates

The phylogenetic analyses of bicarbonate and ammonium transporters in *Symbiodinium* and other dinoflagellates (*K. brevis, L. polyedrum*, *A. carterae*, *C. cohnii*, and *P. minimum)* indicate that these transporters have been duplicated in *Symbiodinium* in a species-specific manner, with many copies sharing high amino acid similarity within a species. This is in line with previous findings in *L. polyedrum* and *C. cohnii* that report on high sequence conservation of genes present in tandem arrays^27^,^55^. The high levels of amino acid sequence conservation of bicarbonate and ammonium transporters as well as tandem array genes implicates functional similarity of duplicated gene copies. This raises interesting possibilities if considered in a broader context such as the paucity of transcription factors^10^,^14^, a canonical promoter structure^2^, and steady-state gene expression profiles in response to environmental changes^5^,^6^,^17^,^18^, but see ref. 56. It has previously been proposed that transcriptional regulation of at least some dinoflagellate genes, such as tandemly arrayed genes, might be controlled through chromatin structure^57^. Following this line of thought, gene duplication could be a suitable mechanism to increase transcript and consequently protein levels of certain genes. Such a model of transcriptional regulation would also provide an explanation for the observation that transcriptional differences between different *Symbiodinium* clades appear to be fixed regardless of the environmental condition^18^.

## Conclusions

Our comparative analyses of three *Symbiodinium* species revealed several genomic features that define this genus and are likely to contribute to their success as widespread endosymbionts. Our results corroborate the prevalence of non-canonical splice-sites and a tendency towards unidirectionality of gene orientation in dinoflagellates. We identified a surprisingly large repertoire of proteins involved in molecule transfer in dinoflagellates, but also highlight enrichment of domains involved in the transport of carbon and nitrogen in the *Symbiodinium* lineage. We also find evidence for substantial differences of these domains between *Symbiodinium* species, which may provide a genomic basis to explain physiological differences and contribute to host-symbiont specificity. The large amount of intraspecific gene duplications in *Symbiodinium* putatively represents an alternate mechanism to increase transcript and protein levels in the absence of strong transcriptional control of gene expression.

## Methods

### Organism and isolation of genomic DNA

*S. microadriaticum* (CCMP2467) cells were obtained from Bigelow National Center for Marine Algae and Microbiota (NCMA) and spread on an f/2 agar plate enriched with antibiotics as described by ref. 58. Six separate colonies were picked and typed using the ITS2 primer pair described in ref. 59. All six colonies were identified as *S. microadriaticum* strain CCMP2467 and a single colony was selected as the source for all subsequent experiments. In order to minimize contamination, cultures were frequently treated with a mix of 10 antibiotics^60^. For DNA extractions, *Symbiodinium* cells were grown in f/2 medium without silica at 26 °C and 80 μmol quanta m^−2^ s^−1^ and harvested in the exponential growth phase (<5 × 10^5^ cells/ml). Briefly, cells were pelleted by centrifugation for 5 min at 3,000 g in a swinging bucket rotor. The cell pellet was washed twice by resuspension in MilliQ water and repeated centrifugation. Subsequently, the cell pellet was snap frozen in liquid nitrogen and transferred to a pre-chilled mortar. Approximately 100 μl of 0.1 mm silica beads were added as grinding aid. The cell pellet was ground to a fine powder. The homogenized cells and beads were transferred to a 50 ml tube and subjected to an RNase treatment for 30 min at 60 °C, followed by a Proteinase K treatment (4 hs at 37 °C) and subsequently extracted using two successive rounds of chloroform extraction, precipitation, and re-extraction with CTAB. The DNA was subjected to two rounds of CTAB extraction to reduce the amount of unwanted polysaccharides in the extraction. The extracted DNA was quantified using a Nanodrop-2000 (Thermo Scientific) and a Qubit (Invitrogen). A 0.8% agarose gel was used to quantify RNA/polysaccharide contamination and to verify that the DNA was of high molecular weight.

### Estimation of genome size

For an estimation of the *S. microadriaticum* genome size, we measured its nuclear DNA content using fluorescence-activated cell sorting (FACS) and the small sea anemone *Aiptasia* as an internal control of known genome size (2C DNA content: 0.53 pg or ~520 Mbp^45^). Nuclei extraction and staining for *S. microadriaticum* and the internal control were performed using the Partec CyStainPI absolute T kit (Partec #05-5023) following the manufacturer’s protocol and the fluorescence signals were measured with a BD FACSCanto II cell analyzer (BD Bioscience) (Supplemental Information, Fig. S1). The reported measurement for *S. microadriaticum* reflects the 1C genome content as *Symbiodinium* is reported to be haploid in culture^61^.

### Genome sequencing

A total of 280 Gb sequence data were generated from the 15 sequence libraries with different fragment sizes (8 short insert size libraries ranging from 200 bp to 1,375 bp, 6 long insert size libraries ranging from 3 kb to 10 kb, 1 fosmid library with ~40 kb insert size) (Supplemental Information, Table S6). All sequence libraries were produced using the Illumina TruSeq DNA kits for paired-end or mate-pair libraries according to the manufacturer’s instructions and sequenced on the Illumina HiSeq platform at the KAUST Bioscience Core Facility (KAUST, Thuwal, KSA) (Supplemental Information, Table S6). MP02 and MP03 were produced and sequenced by GATC-Biotech (Konstanz, Germany). An additional fosmid library was generated by Lucigen (Middleton, WI, USA) and a corresponding mate-pair sequencing library based on 255,000 clones was produced and sequenced on 1 lane of the Illumina HiSeq platform at the KAUST Bioscience Core Facility (KAUST, Thuwal, KSA). All raw sequences have been deposited in the NCBI Sequence Read Archive (SRA) as FASTQ and are accessible under the accession number PRJNA292355 (genomic reads).

### Genome assembly

Reads with more than 5% ambiguous bases (represented by the letter N) or polyA structure contents were discarded. Furthermore, we removed reads with 60% low-quality bases (base quality <5) for the short insert-size libraries and reads with 30% low-quality bases for the long insert-size libraries. Low-quality ends were trimmed directly. This resulted in 2,728,199,233 filtered raw reads providing ~170x coverage that were used for the assembly process. For the assembly, filtered raw reads were pre-assembled using SOAPdenovo2 (Version r240)^62^ and filtered raw reads were mapped against the pre-assembly to determine library insert sizes. For the final assembly, filtered raw reads were first assembled using ALLPATHS-LG (release 47998)^63^ with the default options. The assembly was subjected to two consecutive runs of gap closing using GapCloser (version 1.12-r6)^62^. This gap-closed genome was scaffolded using SSPACE (version 1.2) and the sequencing data from a 40 kb fosmid library based on 255,000 clones. The resulting scaffolds were further scaffolded using L_RNA_Scaffolder^64^ and transcriptome data from ref. 17. The resulting assembly was subjected to two further iterations of gap closing using GapCloser. Assembly statistics were calculated as defined in ref. 65. *The S. microadriaticum* genome is accessible at http://smic.reefgenomics.org^66^ and at NCBI under the accession number PRJNA292355. The genome assembly, gene models, and protein models described in this study are available for download at http://smic.reefgenomics.org/download. A JBrowse genome browser is available at http://smic.reefgenomics.org/jbrowse.

### Identification and removal of contaminating sequences

In order to remove contaminating sequences that were likely to be of bacterial or viral origin, we conducted BLASTN searches against four databases: complete bacterial genomes (ftp://ftp.ncbi.nih.gov/genomes/Bacteria/all.fna.tar.gz), draft bacterial genomes (ftp://ftp.ncbi.nih.gov/genomes/Bacteria_DRAFT/), complete viral genomes (ftp://ftp.ncbi.nih.gov/genomes/Viruses/all.fna.tar.gz) databases from NCBI, and the viral database PhAnToMe (http://phantome.org). As the lengths of the query and hit sequences were up to hundreds of Kb, we used a combination of cutoffs (total bit score >1000, e-value ≤ 10^−20^) to identify scaffolds with significant similarities to sequences in the databases. In total, 1,851 scaffolds – with a median length of 1,628 bp and a combined length of 3.84 Mbp – had over 50% of their non-N sequences being significantly similar to bacterial or viral sequences. These scaffolds were subsequently removed from the final assembly (Dataset S1.7).

### Annotation of repetitive elements

We used RepeatScout^67^ for *de novo* annotation of repetitive elements in the genome assembly using an l-mer size of 16 bp. Using the default settings, a total of 5,622 distinct repeat motifs were identified that occurred ≥10 times. Both, the *de novo* annotated repeat motifs identified by RepeatScout^67^ and a set of known eukaryotic TEs from RepBase (May 2014 release) were then used to locate and annotate the repetitive elements in the assembled *S. microadriaticum* genome using RepeatMasker^68^ (Supplemental Information, Table S7).

### Reference transcriptome sequencing and assembly

*S. microadriaticum* cultures in exponential growth phase were subjected to four different treatments including 4 hs at 36 °C, 4 hs at 16 °C, dark stress (maintained in darkness and harvested at noon), and regular culture conditions (harvested at noon). All treatments were performed in triplicates with the exception of 36 °C, which was performed in duplicate. Cells were harvested as described for the genomic DNA extraction, however, homogenated cells were transferred to microtubes and RNA extracted using the Qiagen Plant RNeasy kit according to the manufacturer’s instructions. RNA samples were quantified using a Qubit (Invitrogen, Carlsbad, CA, USA) and RNA quality was confirmed using a Bioanalyzer RNA NanoChip (Agilent, Santa Clara, CA, USA). Strand-specific RNAseq sequencing libraries were constructed using the NEBNext Ultra Directional RNA Library Prep Kit for Illumina (New England Biolabs, Ipswitch, MA, USA). A total of 3 lanes were sequenced on the Illumina HiSeq platform resulting in 757 million reads. All libraries were trimmed using Trimmomatic^41^ version 0.32 to remove adapters, primers, and low quality ends (Phred score < 30) from reads; reads shorter than 35 bp were removed. PhiX reads were removed using Bowtie2^28^, possible PCR duplicates were removed with PRINSEQ-lite^25^ version 0.20.3, then all libraries were merged and error correction was carried out using ErrorCorrectReads.pl (from ALLPATHS-LG). The resulting library was assembled *de novo* using Trinity (release 20140413)^69^ with strand-specific parameters (--SS_lib_type RF –min_kmer_cov 5 –normalize_reads). The transcriptome reference assembly contains 58,592 transcripts representing 41,679 putative loci (http://smic.reefgenomics.org/download).

### Gene models

Gene models in the *S. microadriaticum* genome were built using the transcriptome assembly and *ab initio* prediction based on selected and refined gene structure models that were annotated with PASA^70^. First, all transcripts of the reference transcriptome (n = 58,592 transcripts, representing 41,679 distinct gene loci) were mapped to the genome assembly using PASA, yielding 22,827 annotated full-length gene structure models. In order to account for putative non-canonical splice sites in the gene structure models, we modified the source code of PASA (http://smic.reefgenomics.org/download). From the set of 22,827 gene structure models, we applied several stringency criteria to filter and obtain a *bona fide* set of gene structure models that could be used as a training gene set for *ab initio* gene prediction using AUGUSTUS (version 3.0.2)^71^,^72^,^73^. The subsequently applied filter steps were: (1) removal of incomplete genes without start and stop codon; (2) removal of genes with less than 3 exons; (3) removal of genes with ambiguous 5′ or 3′ untranslated regions (UTRs); (4) removal of redundant protein sequences in protein clusters as indicated by BLASTP (e-value < 10^−10^), only the longest one was retained in every protein cluster; (5) removal of genes with repeat sequences as indicated by BLASTN alignments to a repeat library generated by RepeatScout (see above). The final set of *bona fide* gene structure models contained 2,957 gene models on which AUGUSTUS was trained in order to predict genes models in the genome assembly using the default training pipeline. To improve the prediction accuracy, we also generated “hints” as supplementary evidence of gene presence and location by mapping all transcripts of the reference transcriptome to the genome assembly using BLAT and the respective scripts provided in AUGUSTUS. For the final *ab initio* gene prediction, the source code of AUGUSTUS was also slightly modified to account for non-canonical exon-intron boundaries (http://smic.reefgenomics.org/download). Finally, the set of *ab initio* genomic gene models was further refined using PASA as described in ref. 45.

### Genome completeness analysis

Genome completeness analyses for all three *Symbiodinium* genomes were initially performed using the CEGMA software^24^, which analyzes the genome to determine the presence of 248 highly conserved eukaryotic genes (CEGs), and resulted in low completeness (<50%). Due to the old evolutionary origin of dinoflagellates that likely predates the split of the three multicellular kingdoms (~1.5–1.9 billion years)^74^,^75^,^76^,^77^, we downloaded the complete list of 458 CEGs used by CEGMA and performed a TBLASTX search against our genomes to identify potential homologs that did not pass the rigorous criteria used by CEGMA. Using a minimum cutoff value of 1e-5, we identified 437 (95.4%), 434 (94.8%), and 383 (83.6%) homologs, of which 9 were only found in *S. microadriaticum,* 6 only in *S. minutum*, and 2 only in *S. kawagutii* (Dataset S1.1).

### Gene orientation analysis

Gene orientation in *S. microadriaticum* was analyzed using a 10-gene window and a 10-gene step. The numbers of orientation changes between every two adjacent genes were counted in every window. For comparative purposes, we performed this analysis on the previously published *S. minutum* and *S. kawagutii* genomes as well as on the genomes of *Plasmodium falciparum*, *Trypanosoma brucei*, *Tetrahymena thermophila*, *Arabidopsis thaliana*, and *Homo sapiens*. A *χ*^*2*^ test was performed to compare the degree of unidirectionality between *S. microadriaticum*, *S. minutum*, *S. kawagutii*, and *T. brucei*, the latter two showed significantly stronger unidirectionality than *S. microadriaticum* and *S. kawagutii*.

### *Symbiodinium* genome composition

In order to determine the putative origins of *Symbiodinium* genes, we performed BLASTP searches of gene models from *S. microadriaticum*, *S. minutum*, and *S. kawagutii* against the NCBI nr database (June 2014). We selected for all hits with e-value ≤ 10^−5^ and then extracted the species that had had the best hit against the *Symbiodinium* gene models. Full taxonomic hierarchy for these organisms were obtained via a Python script that queried Encyclopedia of Life (http://eol.org)^78^ and parsed the resulting JSON reply from the server. Based on the resulting hierarchies, we tallied the number of best hits into seven kingdom/subkingdom-level organismal groups: Archaea, Bacteria, Fungi, Metazoa, Plantae, Protista, and Viruses. For the analysis that allowed for intra-genus matches, we performed pairwise BLASTP searches of *S. microadriaticum*, *S. minutum*, and *S. kawagutii* gene models. For each gene model, if the search produced a higher bitscore (bitscores were used instead of e-values as they are independent of database size) than the corresponding best hit against nr, the best match for that gene model was changed to that of *Symbiodinium*. Chord diagrams were drawn using Circos^79^.

### Functional gene annotation and protein domain enrichment analyses

The final set of predicted proteins was annotated against UniProt (i.e., SwissProt and TrEMBL) and the NCBI nr database to derive gene-based annotations (Dataset S1.8). GO terms were assigned to the gene models as described in ref. 42. Briefly, BLASTP searches of all genomic protein models were successively carried out against SwissProt and TrEMBL databases (June 2014 release). GO terms associated with SwissProt and TrEMBL hits were subsequently obtained from UniProt-GOA (July 2014 release)^80^. If the best-scoring hit of the BLASTP search did not yield any GO annotation, further hits (up to 20 hits, e-value ≤ 10^−5^) were considered, and the best-scoring hit with available GO annotation was used. If none of the SwissProt hits had GO terms associated with them, the TrEMBL database was queried using the same approach. Using this procedure, 22,340 genes (45.5% of the 49,109 gene models) were annotated and had at least one GO term associated with them. Of these, 17,275 had GO annotations via SwissProt, while the remaining 5,065 were retrieved from TrEMBL. In total, 15,989 genes featured e-values < 10^−10^, and 9,871 genes had e-values < 10^−20^, indicating that the majority of the annotations were based on high-confidence alignments to the SwissProt and TrEMBL databases. Proteins that had no matches to either database were subjected to an additional search against the NCBI nr database (e-value ≤ 10^−5^). An additional 2,270 proteins were annotated this way. 24,499 proteins (49.9%) had no hits to any of the three databases – the large fraction of unannotated genes is most likely due to the dearth of dinoflagellate genes in any of these three databases. This procedure was repeated for the *S. minutum* and *S. kawagutii* gene models to eliminate potential biases stemming from the use of different annotation pipelines (Dataset S1.9 and Dataset S1.10).

In order to identify enrichments of protein domains and associated molecular functions encoded in the genomic protein sets of *S. microadriaticum*, *S. minutum,* and *S. kawagutii*, we also annotated the genomic proteins sets of 16 eukaryote reference genomes including *Plasmodium falciparum*, *Tetrahymena thermophila*, *Guillardia theta*, *Emiliania huxleyi*, *Thalassiosira pseudonana*, *Trypanosoma brucei gambiense*, *Chlamydomonas reinhardtii*, and *Capsaspora owczarzaki* as well as the higher eukaryotes *Arabidopsis thaliana, Trichoplax adhaerens*, *Amphimedon queenslandica*, *Stylophora pistillata*, *Lottia gigantea*, *Caenorhabditis elegans*, *Daphnia pulex*, and *Homo sapiens* (Supplemental Information, Table S4) against the Pfam database (version 27) using HMMER^81^ (version 3.1b1) (Dataset S1.2). Domain counts were normalized to the total domain count in each species and expanded protein domain families in *S. microadriaticum, S. minutum*, and *S. kawagutii* were identified with a Fisher’s exact test comparing in-group counts (i.e. *S. microadriaticum*, *S. minutum*, or *S. kawagutii*) to average counts in the outgroups (all other species). Obtained *p*-values were corrected with the Benjamini-Hochberg FDR correction^82^, and z-scores were calculated according to the formula z = (x − u)/s, whereby ‘x’ is the domain ratio, ‘u’ the mean of all domain ratios over all species, and ‘s’ the standard deviation across domain ratios over all species considered. The z-scores from the domain enirchment analyses were visualized using R’s heatmap.2 function (from package gplots)^83^.

We repeated the same analysis using transcriptome data available for *S. microadriaticum* strain KB8^14^, *S. minutum* Mf1.05b^14^, *S. kawagutii*^11^, and the dinoflagellates *K. brevis*^26^, *L. polyedrum*^27^, *Amphidinium carterae*^28^, *Crypthecodinium cohnii*^28^, and *Prorocentrum minimum*^28^ (Dataset S1.3) in order to determine whether the above identified domains were generally enriched in dinoflagellates or unique to *Symbiodinium*. To remove putative isoforms that would artificially inflate the number of domains, we first clustered the transcripts using CD-HIT-EST^84^. For each transcriptome we clustered the transcripts at 90% nucleotide identity resulting in 58,707 transcripts for *S. microadriaticum* KB8, 52,240 for *S. minutum* Mf1.05b, 47,281 for *S. kawagutii*, 83,135 for *K. brevis* SP1, 116,534 for *L. polyedrum*, 29,340 for *Amphidinium carterae,* 33,155 for *Crypthecodinium cohnii*, and 52,466 for *Prorocentrum minimum*. For the within-*Symbiodinium* comparison of enriched protein domains and associated molecular functions, we performed a Fisher’s exact test on the domain annotated genomic protein sets of *S. microadriaticum*, *S. minutum* and *S. kawagutii* using a FDR cutoff of <0.01 (Dataset S1.4).

### Phylogenetic analysis of bicarbonate and ammonium transporters

For the phylogenetic analysis of bicarbonate transporters (PF00955 HCO3_cotransp) and ammonium transporters (PF00909 Ammonium_transp), we extracted protein sequences from all putative genes encoded in the genomes of *S. microadriaticum, S. minutum*, and *S. kawagutii.* Extracted sequences were validated for their putative function by aligning them against a set of reference sequences from bacteria, protists, plants, and animals to verify the presence of conserved amino acid positions. In addition, we extracted homologous sequences from the transcriptomes of *Symbiodinium* species from Clade A, B, C, and D^16^ as well as from the dinoflagellates *K. brevis* SP1, *L. polyedrum*, *Amphidinium carterae*, *Crypthecodinium cohnii*, and *Prorocentrum minimum*. For phylogenetic reconstruction, we aligned the respective sequences using MUSCLE^85^ and trimmed the resulting alignments using trimAl v1.4.1^86^, employing the -automated1 function optimized for maximum-likelihood phylogenetic trees. The best evolutionary model for each of the trimmed alignments was empirically tested using ProtTest3^87^ – LG + G was the most suitable model for PF00955 HCO3_cotransp sequences. The model BLOSUM62 + G was found to perform best for sequences harboring PF00909 Ammonium_transp domain. The alignments were subsequently constructed using RAxML v8.2.0^88^ with 1,000 bootstraps (-x 12345 -p 12345 -N 1000 -f a).

### Availability of supporting data

The genome assembly, gene models, and protein models described in this study are available for download at http://smic.reefgenomics.org/download. A JBrowse genome browser is available at http://smic.reefgenomics.org/jbrowse. Customized PASA and AUGUSTUS scripts for gene calling are available at http://smic.reefgenomics.org/download. A BLAST server for the *Symbiodinium microadriaticum* genome is available at http://smic.reefgenomics.org/blast/. All data reported in the manuscript are deposited at NCBI under the accession number PRJNA292355.

## Additional Information

**How to cite this article**: Aranda, M. *et al*. Genomes of coral dinoflagellate symbionts highlight evolutionary adaptations conducive to a symbiotic lifestyle. *Sci. Rep.* **6**, 39734; doi: 10.1038/srep39734 (2016).

**Publisher's note:** Springer Nature remains neutral with regard to jurisdictional claims in published maps and institutional affiliations.

## Supplementary Material

Supplementary Information

Supplementary Dataset

## Figures and Tables

**Table 1:** Genomes of *Symbiodinium microadriaticum, Symbiodinium minutum*, and *Symbiodinium kawagutii*.

		*Symbiodinium microadriaticum*	*Symbiodinium minutum*	*Symbiodinium kawagutii*
Genome	Genome file used	v1.0	v1.0 (Jun 2013)	Nov 2015
	Total scaffold length (bp)	808,242,489	609,476,485	935,067,369
	Scaffold N50 (bp)	573,512	125,226	380,908
	Total contig length (bp)	746,043,463	603,743,338	902,990,605
	Contig N50 (bp)	34,883	62,714	47,143
	GC content, N excluded (%)	50.5	43.5	45.5
Genes	Number of genes	49,109	41,925	36,850
	Mean gene length (bp)	12,898	11,961	3,788
	Gene models with EST support (%)	76.3	77.2^	72.8*
Exons	Mean coding region length (bp)	2,389	2,027	1,041
	Number of exons per gene	21.8	19.9	4.1
	Mean length (bp)	109.5	101.7	255.7
	Total length (Mb)	117.3	85.0	38.4
Introns	Genes with introns (%)	98.2	95.4	64.1
	Mean length (bp)	504.7	516.6	893.4
	Total length (Mb)	516.1	410.1	101.2
	Predominant first two nucleotides at donor splice sites	GC/GT/GA	GT/GC/GA	GT/GC
Intergenic	Average length (bp)	3,633	2,253	18,035
	Unidirectional arrangement of genes	Yes	Yes^	Yes

^^^Data from Shoguchi *et al*.^10^.

^*^Data from Lin *et al*.^11^.

## References

[b1] Taylor F. J. R. The biology of dinoflagellates. (1987).

[b2] Lin S. Genomic understanding of dinoflagellates. Res. Microbiol. 162, 551–569, doi: 10.1016/j.resmic.2011.04.006 (2011). 21514379

[b3] Hackett J. D., Anderson D. M., Erdner D. L. & Bhattacharya D. Dinoflagellates: a remarkable evolutionary experiment. Am. J. Bot. 91, 1523–1534, doi: 10.3732/ajb.91.10.1523 (2004). 21652307

[b4] Wisecaver J. H., Brosnahan M. L. & Hackett J. D. Horizontal gene transfer is a significant driver of gene innovation in dinoflagellates. Genome Biol. Evol. 5, 2368–2381, doi: 10.1093/gbe/evt179 (2013). 24259313 PMC3879968

[b5] Moustafa A. . Transcriptome profiling of a toxic dinoflagellate reveals a gene-rich protist and a potential impact on gene expression due to bacterial presence. PLoS One 5, e9688 (2010). 20300646 10.1371/journal.pone.0009688 PMC2837391

[b6] Erdner D. & Anderson D. Global transcriptional profiling of the toxic dinoflagellate Alexandrium fundyense using Massively Parallel Signature Sequencing. BMC Genomics 7, 88 (2006). 16638123 10.1186/1471-2164-7-88 PMC1473201

[b7] Mali P., Esvelt K. M. & Church G. M. Cas9 as a versatile tool for engineering biology. Nat. Methods 10, 957–963, doi: 10.1038/nmeth.2649 (2013). 24076990 PMC4051438

[b8] Hou Y. & Lin S. Distinct gene number-genome size relationships for eukaryotes and non-eukaryotes: gene content estimation for dinoflagellate genomes. PLoS One 4, e6978, doi: 10.1371/journal.pone.0006978 (2009). 19750009 PMC2737104

[b9] LaJeunesse T. C., Lambert G., Andersen R. A., Coffroth M. A. & Galbraith D. W. Symbiodinium (Pyrrhophyta) genome sizes (DNA content) are smallest among dinoflagellates. J. Phycol. 41, 880–886 (2005).

[b10] Shoguchi E. . Draft assembly of the Symbiodinium minutum nuclear genome reveals dinoflagellate gene structure. Curr. Biol. 23, 1399–1408, doi: 10.1016/j.cub.2013.05.062 (2013). 23850284

[b11] Lin S. . The Symbiodinium kawagutii genome illuminates dinoflagellate gene expression and coral symbiosis. Science 350, 691–694, doi: 10.1126/science.aad0408 (2015). 26542574

[b12] Trench R. K. Microalgal-invertebrate symbioses: A review. Endocyt. Cell Res. 9, 135–175 (1993).

[b13] Parkinson J. E. . Gene Expression Variation Resolves Species and Individual Strains among Coral-Associated Dinoflagellates within the Genus Symbiodinium. Genome Biology and Evolution 8, 665–680, doi: 10.1093/gbe/evw019 (2016). 26868597 PMC4824173

[b14] Bayer T. . Symbiodinium transcriptomes: genome insights into the dinoflagellate symbionts of reef-building corals. PLoS One 7, e35269, doi: 10.1371/journal.pone.0035269 (2012). 22529998 PMC3329448

[b15] Ladner J. T., Barshis D. J. & Palumbi S. R. Protein evolution in two co-occurring types of Symbiodinium: an exploration into the genetic basis of thermal tolerance in Symbiodinium clade D. BMC Evol. Biol. 12, 217, doi: 10.1186/1471-2148-12-217 (2012). 23145489 PMC3740780

[b16] Rosic N. . Unfolding the secrets of coral-algal symbiosis. ISME J 9, 844–856, doi: 10.1038/ismej.2014.182 (2015). 25343511 PMC4817714

[b17] Baumgarten S. . Integrating microRNA and mRNA expression profiling in Symbiodinium microadriaticum, a dinoflagellate symbiont of reef-building corals. BMC Genomics 14, 704 (2013). 24119094 10.1186/1471-2164-14-704 PMC3853145

[b18] Barshis, D. J., Ladner J. T., Oliver T. A. & Palumbi S. R. Lineage-specific transcriptional profiles of Symbiodinium spp. unaltered by heat stress in a coral host. Mol. Biol. Evol. 31, 1343–1352, doi: 10.1093/molbev/msu107 (2014). 24651035

[b19] Voolstra C. R. . Evolutionary analysis of orthologous cDNA sequences from cultured and symbiotic dinoflagellate symbionts of reef-building corals (Dinophyceae: Symbiodinium). Comparative biochemistry and physiology. Part D, Genomics & proteomics 4, 67–74, doi: 10.1016/j.cbd.2008.11.001 (2009). 20403741

[b20] Lee S. Y., Jeong H. J., Kang N. S., Jang T. Y., Jang S. H. & Lajeunesse T. C. Symbiodinium tridacnidorum sp nov., a dinoflagellate common to Indo-Pacific giant clams, and a revised morphological description of Symbiodinium microadriaticum Freudenthal, emended Trench & Blank. European Journal of Phycology, 50(2), 155–172 (2015).

[b21] Holland B. S., Dawson M. N., Crow G. L. & Hofmann D. K. Global phylogeography of Cassiopea (Scyphozoa: Rhizostomeae): molecular evidence for cryptic species and multiple invasions of the Hawaiian Islands. Mar. Biol. 145, 1119–1128, doi: 10.1007/s00227-004-1409-4 (2004).

[b22] Lajeunesse T. C., Parkinson J. E. & Reimer J. D. A genetics-based description of Symbiodinium minutum sp. nov. and S. psygmophilum sp. nov. (Dinophyceae), two dinoflagellates symbiotic with cnidaria. J. Phycol. 48, 1380–1391, doi: 10.1111/j.1529-8817.2012.01217.x (2012). 27008273

[b23] Pochon X., Montoya-Burgos J. I., Stadelmann B. & Pawlowski J. Molecular phylogeny, evolutionary rates, and divergence timing of the symbiotic dinoflagellate genus Symbiodinium. Mol Phylogenet Evol 38, 20–30, doi: 10.1016/j.ympev.2005.04.028 (2006). 15978847

[b24] Parra G., Bradnam K., Ning Z., Keane T. & Korf I. Assessing the gene space in draft genomes. Nucleic Acids Res. 37, 289–297, doi: 10.1093/nar/gkn916 (2009). 19042974 PMC2615622

[b25] Schmieder R. & Edwards R. Quality control and preprocessing of metagenomic datasets. Bioinformatics 27, 863–864, doi: 10.1093/bioinformatics/btr026 (2011). 21278185 PMC3051327

[b26] Ryan D., Pepper A. & Campbell L. De novo assembly and characterization of the transcriptome of the toxic dinoflagellate Karenia brevis. BMC Genomics 15, 888 (2014). 25306556 10.1186/1471-2164-15-888 PMC4203930

[b27] Beauchemin M. . Dinoflagellate tandem array gene transcripts are highly conserved and not polycistronic. Proc. Natl. Acad. Sci. USA 109, 15793–15798, doi: 10.1073/pnas.1206683109 (2012). 23019363 PMC3465430

[b28] Langmead B. & Salzberg S. L. Fast gapped-read alignment with Bowtie 2. Nat Meth 9, 357–359, doi: 10.1038/nmeth.1923 (2012). PMC3322381 22388286

[b29] Xiang T., Nelson W., Rodriguez J., Tolleter D. & Grossman A. R. Symbiodinium transcriptome and global responses of cells to immediate changes in light intensity when grown under autotrophic or mixotrophic conditions. The Plant Journal 82, 67–80, doi: 10.1111/tpj.12789 (2015). 25664570

[b30] Downs C. A. . Oxidative stress and seasonal coral bleaching. Free Radical Biol. Med. 33, 533–543 (2002). 12160935 10.1016/s0891-5849(02)00907-3

[b31] Weis V. M. Cellular mechanisms of Cnidarian bleaching: stress causes the collapse of symbiosis. J. Exp. Biol. 211, 3059 (2008). 18805804 10.1242/jeb.009597

[b32] Rosic N. N., Pernice M., Dove S., Dunn S. & Hoegh-Guldberg O. Gene expression profiles of cytosolic heat shock proteins Hsp70 and Hsp90 from symbiotic dinoflagellates in response to thermal stress: possible implications for coral bleaching. Cell Stress Chaperon. 16, 69–80, doi: 10.1007/s12192-010-0222-x (2011). PMC3024090 20821176

[b33] Davy S. K., Allemand D. & Weis V. M. Cell Biology of Cnidarian-Dinoflagellate Symbiosis. Microbiol. Mol. Biol. Rev. 76, 229–261, doi: 10.1128/mmbr.05014-11 (2012). 22688813 PMC3372257

[b34] Meyer E. & Weis V. M. Study of Cnidarian-Algal Symbiosis in the “Omics” Age. Biol. Bull. 223, 44–65 (2012). 22983032 10.1086/BBLv223n1p44

[b35] Gordon B. R. & Leggat W. Symbiodinium—Invertebrate Symbioses and the Role of Metabolomics. Mar. Drugs 8, 2546–2568, doi: 10.3390/md8102546 (2010). 21116405 PMC2992991

[b36] Leggat W., Badger M. R. & Yellowlees D. Evidence for an inorganic carbon-concentrating mechanism in the symbiotic dinoflagellate Symbiodinium sp. Plant Physiol. 121, 1247–1256 (1999). 10594111 10.1104/pp.121.4.1247 PMC59491

[b37] Giordano M., Beardall J. & Raven J. A. CO2 concentrating mechanisms in algae: Mechanisms, Environmental Modulation, and Evolution. Annu. Rev. Plant Biol. 56, 99–131, doi: 10.1146/annurev.arplant.56.032604.144052 (2005). 15862091

[b38] Nakajima K., Tanaka A. & Matsuda Y. SLC4 family transporters in a marine diatom directly pump bicarbonate from seawater. Proc. Natl. Acad. Sci. USA 110, 1767–1772, doi: 10.1073/pnas.1216234110 (2013). 23297242 PMC3562803

[b39] Oakley C., Schmidt G. & Hopkinson B. Thermal responses of Symbiodinium photosynthetic carbon assimilation. Coral Reefs 33, 501–512, doi: 10.1007/s00338-014-1130-9 (2014).

[b40] Pernice M. . A single-cell view of ammonium assimilation in coral-dinoflagellate symbiosis. ISME J 6, 1314–1324, http://www.nature.com/ismej/journal/v6/n7/suppinfo/ismej2011196s1.html (2012). 22222466 10.1038/ismej.2011.196 PMC3379633

[b41] Bolger A. M., Lohse M. & Usadel B. Trimmomatic: a flexible trimmer for Illumina sequence data. Bioinformatics 30, 2114–2120, doi: 10.1093/bioinformatics/btu170 (2014). 24695404 PMC4103590

[b42] Liew Y. J. . Identification of microRNAs in the coral Stylophora pistillata. Plos One 9 (2014). 10.1371/journal.pone.0091101 PMC3962355 24658574

[b43] Bhattacharya D. . Comparative genomics explains the evolutionary success of reef-forming corals. eLife 5, e13288, doi: 10.7554/eLife.13288 (2016). 27218454 PMC4878875

[b44] Shinzato C. . Using the Acropora digitifera genome to understand coral responses to environmental change. Nature 476, 320–323, http://www.nature.com/nature/journal/v476/n7360/abs/nature10249.html - supplementary-information (2011). 21785439 10.1038/nature10249

[b45] Baumgarten S. . The genome of Aiptasia, a sea anemone model for coral symbiosis. Proc. Natl. Acad. Sci. USA 112, 11893–11898, doi: 10.1073/pnas.1513318112 (2015). 26324906 PMC4586855

[b46] Gómez F. A quantitative review of the lifestyle, habitat and trophic diversity of dinoflagellates (Dinoflagellata, Alveolata). Syst. Biodivers. 10, 267–275, doi: 10.1080/14772000.2012.721021 (2012).

[b47] Smith E. L. Limiting Factors in Photosynthesis: Light and Carbon Dioxide. J. Gen. Physiol. 22, 21–35 (1938). 19873090 10.1085/jgp.22.1.21 PMC2213731

[b48] Cheng H. . Influence of Co2 Concentrating Mechanism on Photoinhibition in Synechococcus sp. PCC7942 (Cyanophyceae). Phycologia 47, 588–598, doi: 10.2216/07-44.1 (2008).

[b49] Wooldridge S. A. Breakdown of the coral-algae symbiosis: towards formalising a linkage between warm-water bleaching thresholds and the growth rate of the intracellular zooxanthellae. Biogeosciences 10, 1647–1658, doi: 10.5194/bg-10-1647-2013 (2013).

[b50] D’Elia C. F., Domotor S. L. & Webb K. L. Nutrient uptake kinetics of freshly isolated zooxanthellae. Mar. Biol. 75, 157–167, doi: 10.1007/BF00405998 (1983).

[b51] Muscatine L., Falkowski P. G., Dubinsky Z., Cook P. A. & McCloskey L. R. The Effect of External Nutrient Resources on the Population Dynamics of Zooxanthellae in a Reef Coral. “*Proc. R. Soc. Lond., Ser. B*: *Biol. Sci.*” 236, 311–324, doi: 10.1098/rspb.1989.0025 (1989).

[b52] Fabricius K. E. Effects of terrestrial runoff on the ecology of corals and coral reefs: review and synthesis. Mar. Pollut. Bull. 50, 125–146, doi: 10.1016/j.marpolbul.2004.11.028 (2005). 15737355

[b53] Wiedenmann J. . Nutrient enrichment can increase the susceptibility of reef corals to bleaching. Nature Clim. Change 3, 160–164, http://www.nature.com/nclimate/journal/v3/n2/abs/nclimate1661.html - supplementary-information (2013).

[b54] Rädecker N., Pogoreutz C., Voolstra C. R., Wiedenmann J. & Wild C. Nitrogen cycling in corals: the key to understanding holobiont functioning? Trends Microbiol. 23, 490–497, 10.1016/j.tim.2015.03.008 (2015). 25868684

[b55] Mendez G. S., Delwiche C. F., Apt K. E. & Lippmeier J. C. Dinoflagellate Gene Structure and Intron Splice Sites in a Genomic Tandem Array. J. Eukaryot. Microbiol. 62, 679–687, doi: 10.1111/jeu.12230 (2015). 25963315 PMC5032977

[b56] Levin R. A. . Sex, Scavengers, and Chaperones: Transcriptome Secrets of Divergent Symbiodinium Thermal Tolerances. Molecular Biology and Evolution, doi: 10.1093/molbev/msw119 (2016). PMC7297275 27738273

[b57] Wisecaver J. H. & Hackett J. D. Dinoflagellate Genome Evolution. Annu. Rev. Microbiol. 65, 369–387, doi: 10.1146/annurev-micro-090110-102841 (2011). 21682644

[b58] Soffer N., Gibbs P. & Baker A. Practical applications of contaminant-free Symbiodinium cultures grown on solid media. Proc. 11th International Coral Reef Symposium, 159–163 (2008).

[b59] LaJeunesse T. C. & Trench R. K. Biogeography of two species of Symbiodinium (Freudenthal) inhabiting the intertidal sea anemone Anthopleura elegantissima (Brandt). Biol. Bull. 199, 126–134 (2000). 11081711 10.2307/1542872

[b60] Polne-Fuller M. A novel technique for preparation of axenic cultures of Symbiodinium (Pyrrophyta) through selective digestion by amoeba. J. Phycol. 27, 552–554, doi: 10.1111/j.0022-3646.1991.00552.x (1991).

[b61] Santos S. R. & Coffroth M. A. Molecular Genetic Evidence that Dinoflagellates Belonging to the Genus Symbiodinium Freudenthal Are Haploid. Bio. Bull. 204, 10–20 (2003). 12588740 10.2307/1543491

[b62] Luo R. . SOAPdenovo2: an empirically improved memory-efficient short-read de novo assembler. GigaScience 1, 18 (2012). 23587118 10.1186/2047-217X-1-18 PMC3626529

[b63] Gnerre S. . High-quality draft assemblies of mammalian genomes from massively parallel sequence data. Proc. Natl. Acad. Sci. USA 108, 1513–1518, doi: 10.1073/pnas.1017351108 (2011). 21187386 PMC3029755

[b64] Xue W. . L_RNA_scaffolder: scaffolding genomes with transcripts. BMC Genomics 14, 604 (2013). 24010822 10.1186/1471-2164-14-604 PMC3846640

[b65] Bradnam K. R. . Assemblathon 2: evaluating de novo methods of genome assembly in three vertebrate species. GigaScience 2, 10–10, doi: 10.1186/2047-217X-2-10 (2013). 23870653 PMC3844414

[b66] Liew Y. J., Aranda M. & C. R. V. reefgenomics.org-a repository for marine genomics data. Database Accepted (2016). 10.1093/database/baw152 PMC5199144 28025343

[b67] Price A. L., Jones N. C. & Pevzner P. A. De novo identification of repeat families in large genomes. Bioinformatics 21, i351–i358, doi: 10.1093/bioinformatics/bti1018 (2005). 15961478

[b68] Smit A. F. A., Hubley R. & Green P. J. *RepeatMasker Open-3.0*, http://www.repeatmasker.org (1996–2010).

[b69] Haas B. J. . De novo transcript sequence reconstruction from RNA-Seq: reference generation and analysis with Trinity. Nat. Protoc. 8, 10.1038/nprot.2013.1084, doi: 10.1038/nprot.2013.084 (2013). PMC3875132 23845962

[b70] Haas B. J. . Improving the Arabidopsis genome annotation using maximal transcript alignment assemblies. Nucleic Acids Res. 31, 5654–5666 (2003). 14500829 10.1093/nar/gkg770 PMC206470

[b71] Stanke M. & Waack S. Gene prediction with a hidden Markov model and a new intron submodel. Bioinformatics 19 Suppl 2, ii215–225 (2003). 14534192 10.1093/bioinformatics/btg1080

[b72] Stanke M. . AUGUSTUS: ab initio prediction of alternative transcripts. Nucleic Acids Res. 34, W435–439, doi: 10.1093/nar/gkl200 (2006). 16845043 PMC1538822

[b73] Stanke M., Steinkamp R., Waack S. & Morgenstern B. AUGUSTUS: a web server for gene finding in eukaryotes. Nucleic Acids Res. 32, W309–312, doi: 10.1093/nar/gkh379 (2004). 15215400 PMC441517

[b74] Nei M., Xu P. & Glazko G. Estimation of divergence times from multiprotein sequences for a few mammalian species and several distantly related organisms. Proc. Natl. Acad. Sci. USA 98, 2497-2502 (2001). 11226267 10.1073/pnas.051611498 PMC30166

[b75] Hedges S. B. & Kumar S. The timetree of life. (Oxford University Press, 2009).

[b76] Hedges S., Blair J., Venturi M. & Shoe J. A molecular timescale of eukaryote evolution and the rise of complex multicellular life. BMC Evol. Biol. 4, 2 (2004). 15005799 10.1186/1471-2148-4-2 PMC341452

[b77] Parfrey L. W., Lahr D. J. G., Knoll A. H. & Katz L. A. Estimating the timing of early eukaryotic diversification with multigene molecular clocks. Proc. Natl. Acad. Sci. USA 108, 13624–13629, doi: 10.1073/pnas.1110633108 (2011). 21810989 PMC3158185

[b78] Parr C. S. . The Encyclopedia of Life v2: Providing Global Access to Knowledge About Life on Earth. Biodivers Data J. e1079, doi: 10.3897/BDJ.2.e1079 (2014). 24891832 PMC4031434

[b79] Krzywinski M. . Circos: An information aesthetic for comparative genomics. Genome Res. 19, 1639–1645, doi: 10.1101/gr.092759.109 (2009). 19541911 PMC2752132

[b80] Dimmer E. . The UniProt-GO Annotation database in 2011. Nucleic Acids Res. 40, D565–D570 (2012). 22123736 10.1093/nar/gkr1048 PMC3245010

[b81] Mistry J., Finn R. D., Eddy S. R., Bateman A. & Punta M. Challenges in homology search: HMMER3 and convergent evolution of coiled-coil regions. Nucleic Acids Research 41, e121, doi: 10.1093/nar/gkt263 (2013). 23598997 PMC3695513

[b82] Benjamini Y. & Hochberg Y. Controlling the False Discovery Rate: A Practical and Powerful Approach to Multiple Testing. Journal of the Royal Statistical Society. Series B, Statistical methodology 57, 289–300 (1995).

[b83] Warnes G. R. . gplots: Various R programming tools for plotting data. R package version 2 (2009).

[b84] Li W. & Godzik A. Cd-hit: a fast program for clustering and comparing large sets of protein or nucleotide sequences. Bioinformatics 22, 1658–1659 (2006). 16731699 10.1093/bioinformatics/btl158

[b85] Edgar R. C. MUSCLE: multiple sequence alignment with high accuracy and high throughput. Nucleic Acids Res. 32, 1792–1797, doi: 10.1093/nar/gkh340 (2004). 15034147 PMC390337

[b86] Capella-Gutierrez S., Silla-Martinez J. M. & Gabaldon T. trimAl: a tool for automated alignment trimming in large-scale phylogenetic analyses. Bioinformatics 25, 1972–1973, doi: 10.1093/bioinformatics/btp348 (2009). 19505945 PMC2712344

[b87] Darriba D., Taboada G. L., Doallo R. & Posada D. ProtTest 3: fast selection of best-fit models of protein evolution. Bioinformatics 27, 1164–1165, doi: 10.1093/bioinformatics/btr088 (2011). 21335321 PMC5215816

[b88] Stamatakis A. RAxML version 8: a tool for phylogenetic analysis and post-analysis of large phylogenies. Bioinformatics 30, 1312–1313, doi: 10.1093/bioinformatics/btu033 (2014). 24451623 PMC3998144

