# Supplementary material for: Genomes of coral dinoflagellate symbionts highlight evolutionary adaptations conducive to a symbiotic lifestyle
Source: Sci Rep. 2016 Dec 22;6:39734. doi: 10.1038/srep39734 (PMC5177918; doi:10.1038/srep39734)
Supplement: Supplementary Information [file srep39734-s1.pdf]

Genomes of coral dinoflagellate symbionts highlight evolutionary adaptations conducive to a symbiotic lifestyle

Short title: Comparative analysis of coral dinoflagellate genomes

M. Aranda[a], Y. Li[a], Y.J. Liew[a], S. Baumgarten[a], O. Simakov[b], M. C. Wilson[c], J. Piel[c], H. Ashoor, S. Bougouffa[d], V.B. Bajic[d], T. Ryu[e], T. Ravasi[e], T. Bayer[a,f], G. Micklem[g], H. Kim[h], J. Bhak[h], T.C. LaJeunesse[i], C.R. Voolstra[a]

[a] Red Sea Research Center, Division of Biological and Environmental Science and Engineering (BESE), King Abdullah University of Science and Technology (KAUST), Thuwal 23955-6900, Saudi Arabia
[b] Centre for Organismal Studies, Heidelberg University, 69120 Heidelberg, Germany
[c] Institute of Microbiology, Eidgenössische Technische Hochschule Zurich, Vladimir-Prelog-Weg 4, 8093 Zurich, Switzerland
[d] Computational Bioscience Research Center (CBRC), Computer, Electrical and Mathematical Sciences and Engineering Division (CEMSE), King Abdullah University of Science and Technology (KAUST), Thuwal 23955-6900, Saudi Arabia
[e] KAUST Environmental Epigenetics Program (KEEP), Division of Biological and Environmental Sciences and Engineering (BESE), King Abdullah University of Science and Technology (KAUST), Thuwal 23955-6900, Saudi Arabia
[f] GEOMAR Department: Evolutionary Ecology of Marine Fishes, GEOMAR Helmholtz Centre for Ocean Research, Kiel, Germany,
[g] Department of Genetics, University of Cambridge, Cambridge CB2 3EH, UK
[h] Personal Genomics Institute, Genome Research Foundation, Suwon, Republic of Korea
[i] Department of Biology, The Pennsylvania State University, University Park, PA 16802

Corresponding authors

Christian R. Voolstra
Associate Professor of Marine Science
Red Sea Research Center
KAUST University
Thuwal 23955-6900, Saudi Arabia
email: christian.voolstra@kaust.edu.sa
Phone: +966 5 44 7000 87

Manuel Aranda
Assistant Professor of Marine Science
Red Sea Research Center
KAUST University
Thuwal 23955-6900, Saudi Arabia
email: manuel.aranda@kaust.edu.sa
Phone: +966 5 44 7006 61

Supplemental Information

Supplemental Analysis

Comparison of genomes to transcriptomes.

To determine the usability of transcriptomic data as proxy to identify genome-based domain enrichment, we tested how representative *Symbiodinium* (and by extension dinoflagellates) transcriptomes are of their underlying genomes. We found that the transcriptome- and genome-derived domain ratios for all three *Symbiodinium* species were highly significantly correlated (*S. microadriaticum* genome vs. transcriptome $R^2 = 0.74$, p-value $< 10^{-16}$; *S. minutum* genome vs. transcriptome $R^2 = 0.64$, p-value $< 10^{-16}$, *S. kawagutii* genome vs. transcriptome $R^2 = 0.58$, p-value $< 10^{-16}$ SI Appendix, Fig. S5). At the same time, we saw inflation of some domains in the transcriptome in comparison to the genome and *vice versa*. While the comparison of genomic and transcriptomic data is problematic in nature it has previously been shown that > 90% of the genes identified in *Symbiodinium* transcriptomes are expressed under any condition tested, indicating that dinoflagellate transcriptomes might serve as a proxy for the prohibitively large underlying genomes.

Supplemental Tables

Table S1. Assembly statistics for the *Symbiodinium microadriaticum* genome assembly

Overview statistics		
	Genome size (FACS)	1.4 Gbp
	Genome size (AllPaths)	1.1 Gbp
	Total size of scaffolds	808,242,489 bp
	Number of scaffolds	9,695
	N50 scaffold length	573,512 bp
	Total size of contigs	746,043,463 bp
	Number of contigs	43,068
	N50 contig length	34,883 bp
	Avg. length of gaps (for gaps of >25 Ns)	1,864
Scaffold statistics		
	Longest scaffold	3,144,590 bp
	Shortest scaffold	891 bp
	Number of scaffolds >1 kb	9,549
	Number of scaffolds >10 kb	2,691
	Number of scaffolds >100 kb	1,652
	Number of scaffolds >1 Mb	136
	Mean scaffold size	83,367 bp
	Median scaffold size	2,325 bp
Contig statistics		
	Longest contig	329,290 bp
	Number of contigs >1 kb	42,859
	Number of contigs >10 kb	20,725
	Number of contigs >100 kb	522
	Number of contigs >1 Mb	0
	Mean contig size	17,322 bp
	Median contig size	9,253 bp

Table S2. Annotation statistics for the *Symbiodinium microadriaticum* gene models

Total number of gene models	49,109	100%
Number of gene models with KEGG annotations	9,570	19%
Number of gene models with PANTHER annotations	19,470	40%
Number of gene models with Pfam domains	18,702	38%
Total number of gene models with associated GO terms based on all UniProt annotations	22,340	45%
Number of gene models with associated GO terms based on SwissProt annotations	17,275	35%
Number of gene models with associated GO terms based on TrEMBL annotations	5,065	10%
Number of gene models annotated against nr database	2,270	5%

Table S3. Number of syntenic blocks between the genomes of *S. microadriaticum* and *S. minutum, S. microadriaticum* and *S. kawagutii,* and *S. minutum* and *S. kawagutii* using MCScanX (-e_value: 1e-5; -match_size = 5).

**syntenic blocks [gene pairs]**	**Smic_vs_Smin**	**Smic_vs_Skav**	**Smin_vs_Skav**
<=10	349	166	222
<=15	28	18	32
>15	4	2	3
Total	381	186	257

Table S4. Reference genomic proteins sets used for Pfam domain enrichment analyses.

Protozoans	Reference
Plasmodium falciparium (Pfalciparum3D7)	http://plasmodb.org
Guillardia theta	UniProt Proteome Database
Emiliania huxleyi	UniProt Proteome Database
Thalassiosira pseudonana	UniProt Proteome Database
Trypanosoma brucei gambiense (TbruceigambienseDAL972)	http://tritrypdb.org
Tetrahymena thermophylia	http://ciliate.org
Capsaspora owczarzaki	http://www.ncbi.nlm.nih.gov/nuccore/514340846
**Metazoans**	
Trichoplax adherens	FilteredModels2 from JGI (August 2007)
Amphimedon queenslandica	Aqu1 models (August 2008)
Stylophora pistillata	Liew et al 2014
Lottia gigantea	Limpet proteome from JGI (Lotgi1@shake, FilteredModels1 table) (May 2007)
Caenorhabditis elegans	Wormbase release WS164
Daphnia pulex	FilteredModels8 from JGI (September 2007)
Homo sapiens	NCBI 36 from Ensembl 41
**Plants**	
Chlamydomonas reinhardtii	http://phytozome.jgi.doe.gov
Arabidopsis thaliana	TAIR10 from www.arabidopsis.org/

Table S5. Significantly enriched domains in *Symbiodinium* in comparison to 16 reference eukaryotic protein sets and 5 dinoflagellate transcriptomes. *Symbiodinium*-specific category highlights 40 domains specifically enriched in *Symbiodinium* that were identified in both comparisons.

***Symbiodinium* vs. Eukaryotes**	***Symbiodinium* vs. Dinoflagellates**	***Symbiodinium*-specific**
PF00016_RuBisCO_large	PF00016_RuBisCO_large	PF00016_RuBisCO_large
PF00091_Tubulin	PF00629_MAM	PF00091_Tubulin
PF00112_Peptidase_C1	PF00860_Xan_ur_permease	PF00955_HCO3_cotransp
PF00174_Oxidored_molyb	PF00955_HCO3_cotransp	PF01292_Ni_hydr_CYTB
PF00175_NAD_binding_1	PF01292_Ni_hydr_CYTB	PF08241_Methyltransf_11
PF00254_FKBP_C	PF01453_B_lectin	PF00069_Pkinase
PF00355_Rieske	PF01637_Arch_ATPase	PF01490_Aa_trans
PF00391_PEP-utilizers	PF07690_MFS_1	PF03953_Tubulin_C
PF00782_DSPc	PF07714_Pkinase_Tyr	PF07690_MFS_1
PF00955_HCO3_cotransp	PF13401_AAA_22	PF00173_Cyt-b5
PF01292_Ni_hydr_CYTB	PF13517_VCBS	PF00216_Bac_DNA_binding
PF01341_Glyco_hydro_6	PF17123_zf-RING_11	PF00484_Pro_CA
PF01434_Peptidase_M41	PF12861_zf-ANAPC11	PF00504_Chloroa_b-bind
PF01612_DNA_pol_A_exo1	PF00032_Cytochrom_B_C	PF00648_Peptidase_C2
PF01844_HNH	PF00223_PsaA_PsaB	PF00849_PseudoU_synth_2
PF02338_OTU	PF00421_PSII	PF00909_Ammonium_transp
PF02536_mTERF	PF08309_LVIVD	PF02429_PCP
PF02668_TauD	PF12796_Ank_2	PF03030_H_PPase
PF03033_Glyco_transf_28	PF13637_Ank_4	PF05725_FNIP
PF03441_FAD_binding_7	PF13857_Ank_5	PF12796_Ank_2
PF03600_CitMHS	PF15913_Furin-like_2	PF12847_Methyltransf_18
PF03971_IDH	PF00033_Cytochrome_B	PF13041_PPR_2
PF04324_Fer2_BFD	PF08477_Miro	PF13392_HNH_3
PF04403_PqiA	PF00504_Chloroa_b-bind	PF13517_VCBS
PF04989_CmcI	PF03953_Tubulin_C	PF13540_RCC1_2
PF05183_RdRP	PF00091_Tubulin	PF13578_Methyltransf_24
PF05295_Luciferase_N	PF00484_Pro_CA	PF13857_Ank_5
PF06514_PsbU	PF00651_BTB	PF08309_LVIVD
PF06594_HCBP_related	PF00909_Ammonium_transp	PF13637_Ank_4
PF06839_zf-GRF	PF10563_CdCA1	PF00121_TIM
PF07691_PA14	PF13540_RCC1_2	PF13306_LRR_5
PF08241_Methyltransf_11	PF00022_Actin	PF05221_AdoHcyase
PF08449_UAA	PF00067_p450	PF08242_Methyltransf_12
PF08877_MepB	PF00124_Photo_RC	PF10563_CdCA1
PF13360_PQQ_2	PF00216_Bac_DNA_binding	PF13606_Ank_3
PF13489_Methyltransf_23	PF00432_Prenyltrans	PF13649_Methyltransf_25
PF13659_Methyltransf_26	PF00648_Peptidase_C2	PF13847_Methyltransf_31

PF13884_Peptidase_S74
PF14307_Glyco_tran_WbsX
PF14528_LAGLIDADG_3
PF07857_TMEM144
PF00013_KH_1
PF00026_Asp
PF00027_cNMP_binding
PF00069_Pkinase
PF00090_TSP_1
PF00098_zf-CCHC
PF00128_Alpha-amylase
PF00144_Beta-lactamase
PF00149_Metallophos
PF00150_Cellulase
PF00225_Kinesin
PF00248_Aldo_ket_red
PF00300_His_Phos_1
PF00481_PP2C
PF00501_AMP-binding
PF00550_PP-binding
PF00639_Rotamase
PF00642_zf-CCCH
PF00652_Ricin_B_lectin
PF00654_Voltage_CLC
PF00686_CBM_20
PF00737_PsbH
PF00810_ER_lumen_recept
PF00856_SET
PF00975_Thioesterase
PF01041_DegT_DnrJ_EryC1
PF01062_Bestrophin
PF01490_Aa_trans
PF01501_Glyco_transf_8
PF01529_zf-DHHC
PF01596_Methyltransf_3
PF01699_Na_Ca_ex
PF01716_MSP
PF01755_Glyco_transf_25
PF01764_Lipase_3
PF02230_Abhydrolase_2
PF02233_PNTB
PF02492_cobW
PF02788_RuBisCO_large_N
PF00849_PseudoU_synth_2
PF08241_Methyltransf_11
PF08242_Methyltransf_12
PF12847_Methyltransf_18
PF13041_PPR_2
PF13306_LRR_5
PF13392_HNH_3
PF13578_Methyltransf_24
PF13606_Ank_3
PF13631_Cytochrom_B_N_2
PF13649_Methyltransf_25
PF13847_Methyltransf_31
PF13855_LRR_8
PF16861_Carbam_trans_C
PF02429_PCP
PF05725_FNIP
PF00069_Pkinase
PF00118_Cpn60_TCP1
PF00121_TIM
PF00173_Cyt-b5
PF01490_Aa_trans
PF02867_Ribonuc_red_lgC
PF03030_H_PPase
PF05221_AdoHcyase
PF16861_Carbam_trans_C
PF17123_zf-RING_11
PF08477_Miro

PF02825_WWE
PF03016_Exostosin
PF03092_BT1
PF03109_ABC1
PF03133_TTL
PF03176_MMPL
PF03741_TerC
PF03747_ADP_ribosyl_GH
PF03953_Tubulin_C
PF03959_FSH1
PF04142_Nuc_sug_transp
PF04378_RsmJ
PF04515_Choline_transpo
PF04828_GFA
PF05049_IIGP
PF05050_Methyltransf_21
PF05118_Asp_Arg_Hydrox
PF05517_p25-alpha
PF05637_Glyco_transf_34
PF05721_PhyH
PF06414_Zeta_toxin
PF07683_CobW_C
PF07690_MFS_1
PF08007_Cupin_4
PF08016_PKD_channel
PF09409_PUB
PF10294_Methyltransf_16
PF10436_BCDHK_Adom3
PF12437_GSIII_N
PF13205_Big_5
PF13248_zf-ribbon_3
PF13415_Kelch_3
PF13499_EF-hand_7
PF13532_2OG-FeII_Oxy_2
PF13535_ATP-grasp_4
PF13621_Cupin_8
PF13640_2OG-FeII_Oxy_3
PF13675_PilJ
PF13738_Pyr_redox_3
PF13759_2OG-FeII_Oxy_5
PF14765_PS-DH
PF00023_Ank
PF00036_EF-hand_1

PF00075_RNase_H
PF00109_ketoacyl-synt
PF00145_DNA_methylase
PF00173_Cyt-b5
PF00216_Bac_DNA_binding
PF00226_DnaJ
PF00230_MIP
PF00313_CSD
PF00484_Pro_CA
PF00504_Chloroa_b-bind
PF00520_Ion_trans
PF00644_PARP
PF00648_Peptidase_C2
PF00734_CBM_1
PF00840_Glyco_hydro_7
PF00849_PseudoU_synth_2
PF00890_FAD_binding_2
PF00909_Ammonium_transp
PF01326_PPDK_N
PF01885_PTS_2-RNA
PF02364_Glucan_synthase
PF02429_PCP
PF02801_Ketoacyl-synt_C
PF03030_H_PPase
PF03372_Exo_endo_phos
PF04059_RRM_2
PF05704_Caps_synth
PF05725_FNIP
PF05903_Peptidase_C97
PF08323_Glyco_transf_5
PF08659_KR
PF12314_IMCp
PF12695_Abhydrolase_5
PF12796_Ank_2
PF12847_Methyltransf_18
PF13041_PPR_2
PF13392_HNH_3
PF13517_VCBS
PF13540_RCC1_2
PF13578_Methyltransf_24
PF13604_AAA_30
PF13646_HEAT_2
PF13857_Ank_5

PF02543_Carbam_trans_N
PF00044_Gp_dh_N
PF02690_Na_Pi_cotrans
PF02896_PEP-utilizers_C
PF08309_LVIVD
PF13245_AAA_19
PF13637_Ank_4
PF00005_ABC_tran
PF00012_HSP70
PF00121_TIM
PF00270_DEAD
PF00271_Helicase_C
PF00283_Cytochrom_B559
PF00365_PFK
PF00397_WW
PF00463_ICL
PF00498_FHA
PF00535_Glycos_transf_2
PF00575_S1
PF00588_SpoU_methylase
PF00650_CRAL_TRIO
PF00668_Condensation
PF00698_Acyl_transf_1
PF00916_Sulfate_transp
PF01033_Somatomedin_B
PF01036_Bac_rhodopsin
PF01116_F_bP_aldolase
PF01189_Nol1_Nop2_Fmu
PF01504_PIP5K
PF01522_Polysacc_deac_1
PF01594_UPF0118
PF01740_STAS
PF01795_Methyltransf_5
PF02037_SAP
PF02181_FH2
PF02419_PsbL
PF02518_HATPase_c
PF03071_GNT-I
PF03151_TPT
PF03618_Kinase-PPPase
PF03982_DAGAT
PF03992_ABM
PF06472_ABC_membrane_2

PF07699_GCC2_GCC3
PF08393_DHC_N2
PF11527_ARL2_Bind_BART
PF12780_AAA_8
PF12849_PBP_like_2
PF13365_Trypsin_2
PF13418_Kelch_4
PF13472_Lipase_GDSL_2
PF13639_zf-RING_2
PF13913_zf-C2HC_2
PF14310_Fn3-like
PF14424_Toxin-deaminase
PF02714_RSN1_7TM
PF01788_PsbJ
PF02800_Gp_dh_C
PF04851_ResIII
PF07992_Pyr_redox_2
PF13202_EF-hand_5
PF13306_LRR_5
PF14495_Cytochrom_C550
PF00037_Fer4
PF00097_zf-C3HC4
PF00583_Acetyltransf_1
PF00589_Phage_integrase
PF00903_Glyoxalase
PF01855_POR_N
PF03457_HA
PF04886_PT
PF05221_AdoHcyase
PF06723_MreB_Mbl
PF08017_Fibrinogen_BP
PF08242_Methyltransf_12
PF08627_CRT-like
PF09439_SRPRB
PF10563_CdCA1
PF12146_Hydrolase_4
PF12678_zf-rbx1
PF12681_Glyoxalase_2
PF12831_FAD_oxidored
PF12837_Fer4_6
PF13014_KH_3
PF13187_Fer4_9
PF13237_Fer4_10

PF13374_TPR_10
PF13405_EF-hand_6
PF13423_UCH_1
PF13424_TPR_12
PF13432_TPR_16
PF13434_K_oxygenase
PF13442_Cytochrome_CBB3
PF13508_Acetyltransf_7
PF13606_Ank_3
PF13649_Methyltransf_25
PF13673_Acetyltransf_10
PF13806_Rieske_2
PF13833_EF-hand_8
PF13847_Methyltransf_31
PF13923_zf-C3HC4_2
PF14464_Prok-JAB
PF14531_Kinase-like
PF14542_Acetyltransf_CG
PF14634_zf-RING_5
PF15711_ILEI
PF16113_ECH_2
PF16114_Citrate_bind
PF16363_GDP_Man_Dehyd
PF16499_Melibiase_2
PF16639_Apocytochr_F_N
PF16861_Carbam_trans_C
PF17123_zf-RING_11
PF08477_Miro

Table S6. Sequencing libraries used for genome assembly and gap filling. Library abbreviations are as follows: paired-end (PE), mate-pair (MP), and fosmid libraries (FM).

Library	SRA accession	Targeted insert size	Read # before filtering	Read # after filtering	Assembly	Gap filling
PE04	SRX1438245	975	161,714,372	129,874,065	+	
PE05	SRX1438246	800	94,178,368	78,948,347	+	+
PE07	SRX1438247	525	176,120,320	149,656,500	+	+
PE09	SRX1438249	1375	236,814,098	175,391,970	+	
PE10	SRX1438250	1375	223,336,000	166,112,239	+	
PE13	SRX1438251	200	230,703,186	213,455,375	+	+
PE14	SRX1438252	200	235,739,990	217,849,074	+	+
PE15	SRX1438253	500	287,184,008	254,736,456	+	+
MP02	SRX1438236	3000	441,999,004	401,003,422	+	
MP03	SRX1438238	3000	385,859,256	332,783,326	+	
MP04	SRX1438238	6000	99,328,060	74,146,748	+	
MP05	SRX1438241	7000	80,033,522	66,997,041	+	
MP06	SRX1438242	9000	186,006,370	146,629,029	+	
MP07	SRX1438243	10000	160,220,924	123,706,329	+	
FM01	SRX1438244	40000	217,238,076	196,909,312	+	

Table S7. *S. microadriatricum* repeat content.

Element [a]	Number of occurrences [b]	Number of bp covered [b]
**DNA Transposon**	**169414**	**9299507**
MuDR	7580	448775
EnSpm	80349	3460714
Mariner	7264	502889
Crypton	548	17619
Rehavkus	21	2236
Polinton	7118	469975
Zator	1	70
Sola	21926	1712634
Tc1	5	325
Helitron	6351	540669
ISL2EU	218	12464
Merlin	98	8668
P	5316	254291
hAT	15642	1019180
Kolobok	2532	143456
Harbinger	490	22186
Chapaev	4848	223176
PiggyBac	62	2807
Novosib	5022	246820
Transib	4023	210553
**LTR Retrotransposon**	**21444**	**3472654**
DIRS	2717	475852
ROO	102	5306
Copia	6723	2022223
BEL	180	16004
Gypsy	11722	953269
**Other transposons**	**742572**	**50017724**
Other	742572	50017724
**snRNA**	**235**	**23913**
snRNA	235	23913
**Endogenous Retrovirus**	**4443**	**161688**
ERV	4443	161688
**Other**	**72773**	**12613861**
Satellite	42764	6281999
Other transposons	30009	6331862
**rRNA**	**5716**	**684501**
SSU-rRNA	5301	666285
rRNA	415	18216
**Non-LTR Retrotransposon**	**239029**	**16176009**
Proto	14	956
Penelope	290	21126

Crack	20	888
R1	1043	66727
Nematis	131	4868
CRE	46	10310
RTEX	55	3483
Rex	45	13984
Jockey	2960	174008
L1	175702	10778128
Ingi	37	8794
L2	2060	117988
RTE	607	285833
Randl	149	149068
Nimbus	24	5715
Perere	23	3631
Tad1	6	360
Tx1	2389	126163
SINE	29	875
Neptune	1752	114297
R4	1	77
Daphne	29304	2486081
Poseidon	155	18731
Hero	22	1427
I	18	1261
CR1	22074	1729741
R2	73	51489
tRNA	17	978
**Unclassified** [c]	**45683202**	**45683202**
**Low complexity**	**907361**	**78122968**
Other low complexity	411640	42679795
Simple repeat	495721	35443173

[a] Categories of elements are indicated in bold face.

[b] Bold face indicates subtotals for the respective categories.

[c] Previously described but poorly defined repetitive elements.

Supplemental Figures

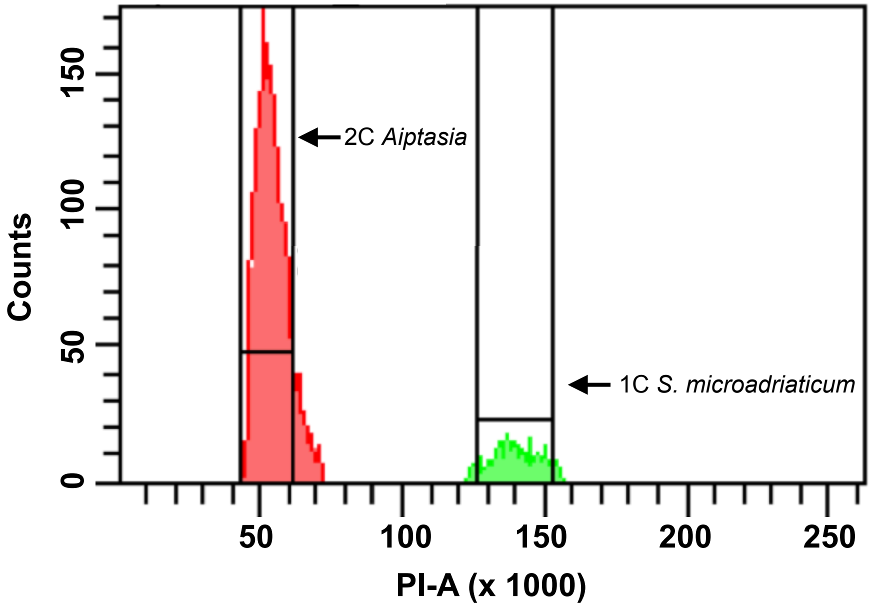

Fig. S1. Estimation of the *S. microadriaticum* genome size. The count profile of the Fluorescence-activated cell sorting (FACS) shows two peaks, the propidium-iodide-stained nuclei of the internal control *Aiptasia* (red) and the nuclei staining of *S. microadriaticum* (green). Given the known *Aiptasia* 2C DNA content of ~520 Mbp, the haploid DNA content of *S. microadriaticum* is estimated to be ~1,400 Mbp.

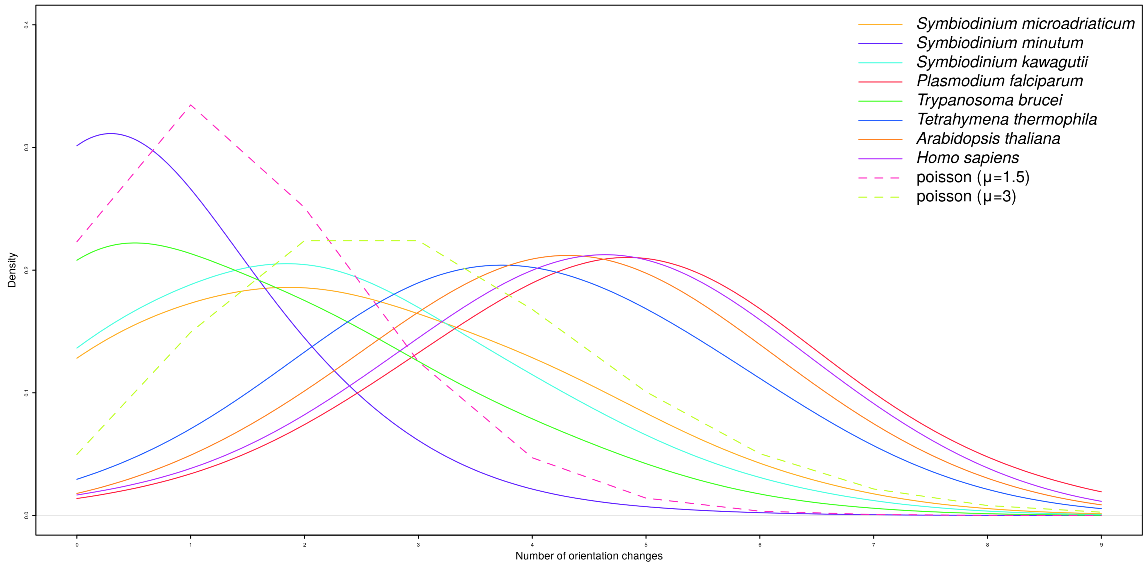

Fig. S2. Distribution of gene orientation changes. Genomes were analyzed using a 10-gene sliding window and a 10-gene step, the numbers of orientation changes between every two adjacent genes were counted in every window. *S. microadriaticum*, *S. minutum*, and *S. kawagutii* show very few orientation changes.

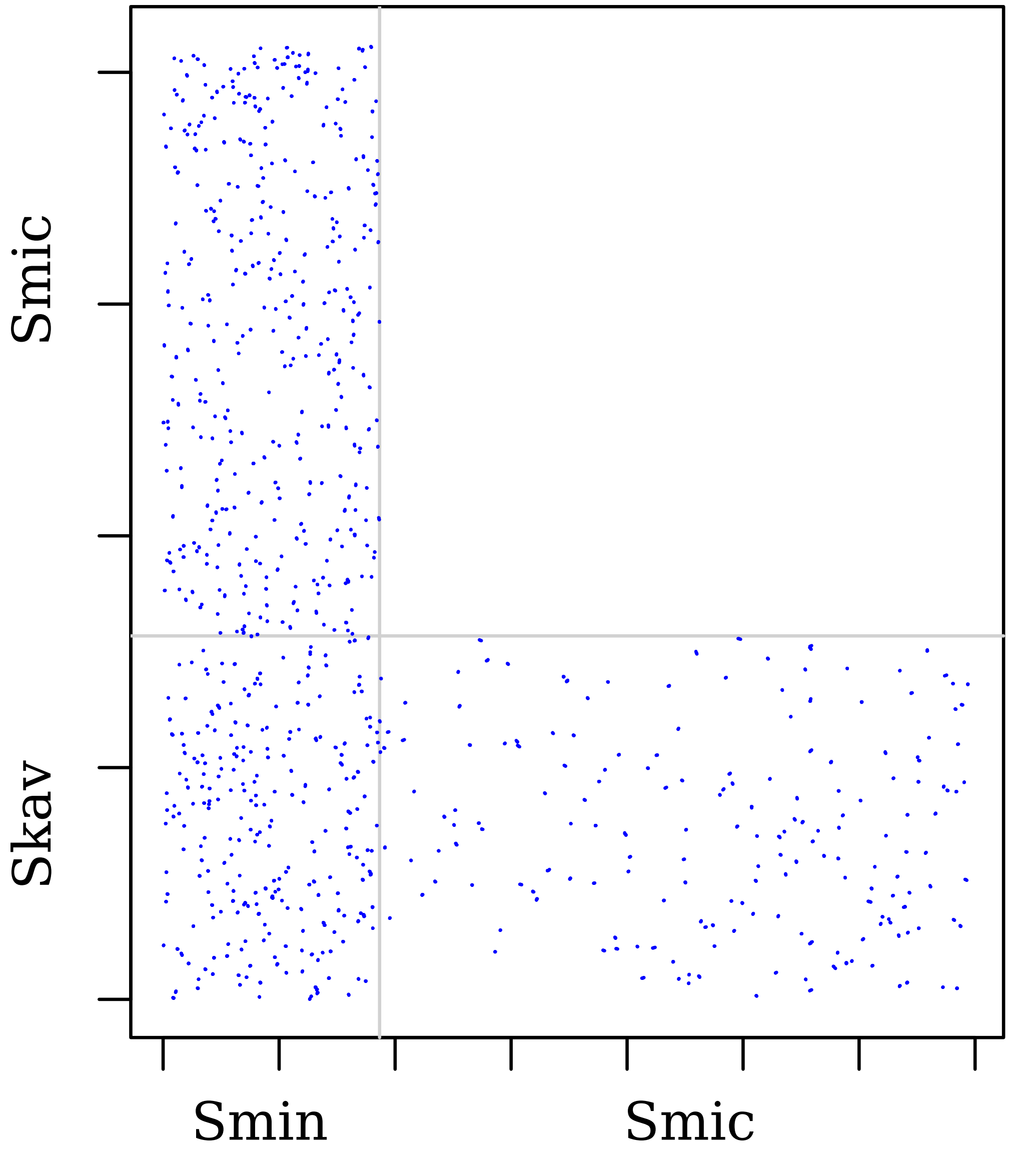

Fig. S3. Dotplot based on gene synteny analyses between the genomes of *S. microadriaticum* (Smic), *S. minutum* (Smin), and *S. kawagutii* (Skav) based on MCScanX and syntenic blocks of 5 or more genes (-e_value: 1e-5; -match_size=5).

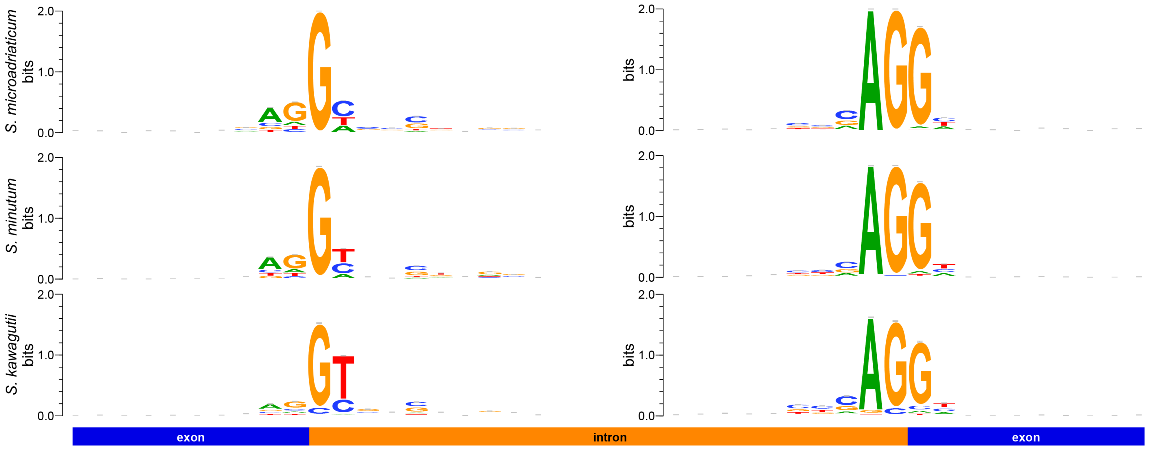

Fig. S4. Non-canonical splice sites in the genomes of *S. microadriaticum*, *S. minutum*, and *S. kawagutii*. While all species displayed non-canonical donor sites, the proportions were quite different: *S. microadriaticum* had 21.9% GA, 52.1% GC, and 26.0% GT; *S. minutum* had 15.3% GA, 35.9% GC and 47.0% GT; *S. kawagutii* had 2.3% GA, 23.2% GC, 65.6% GT. The makeup of the acceptor sites for the three species were very similar, having a non-canonical G immediately following the acceptor splice site AG (96.2% in *S. microadriaticum*, 93.7% in *S. minutum*, and 86.4% in *S. kawagutii*).

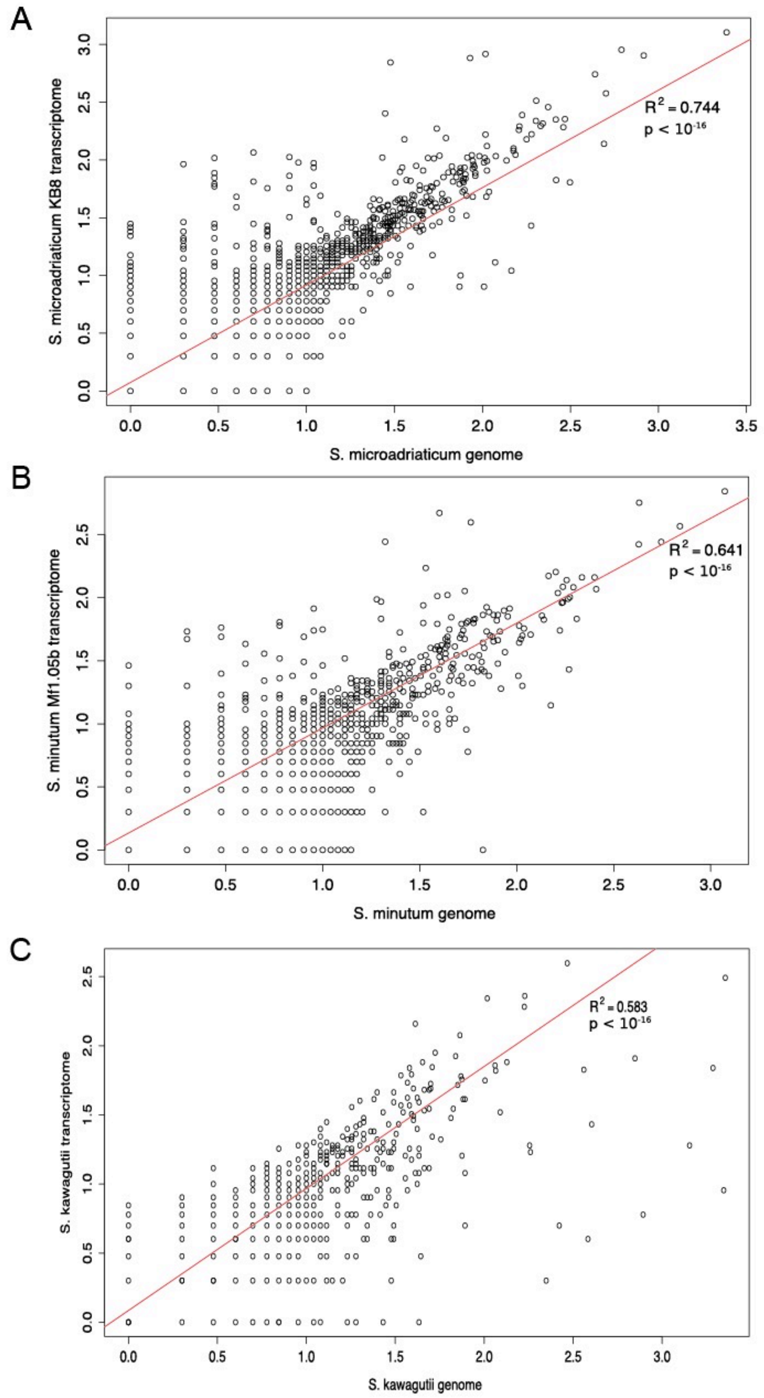

Fig. S5. Correlation of transcriptome- and genome-derived domain ratios for three *Symbiodinium* species. (A) *S. microadriaticum*, (B) *S. minutum*, and (C) *S. kawagutii*. Domain counts were log transformed and plotted using R. Adjusted R-square values as well as the corresponding *p*-values are provided ($p < 10^{-16}$ for all analyses).

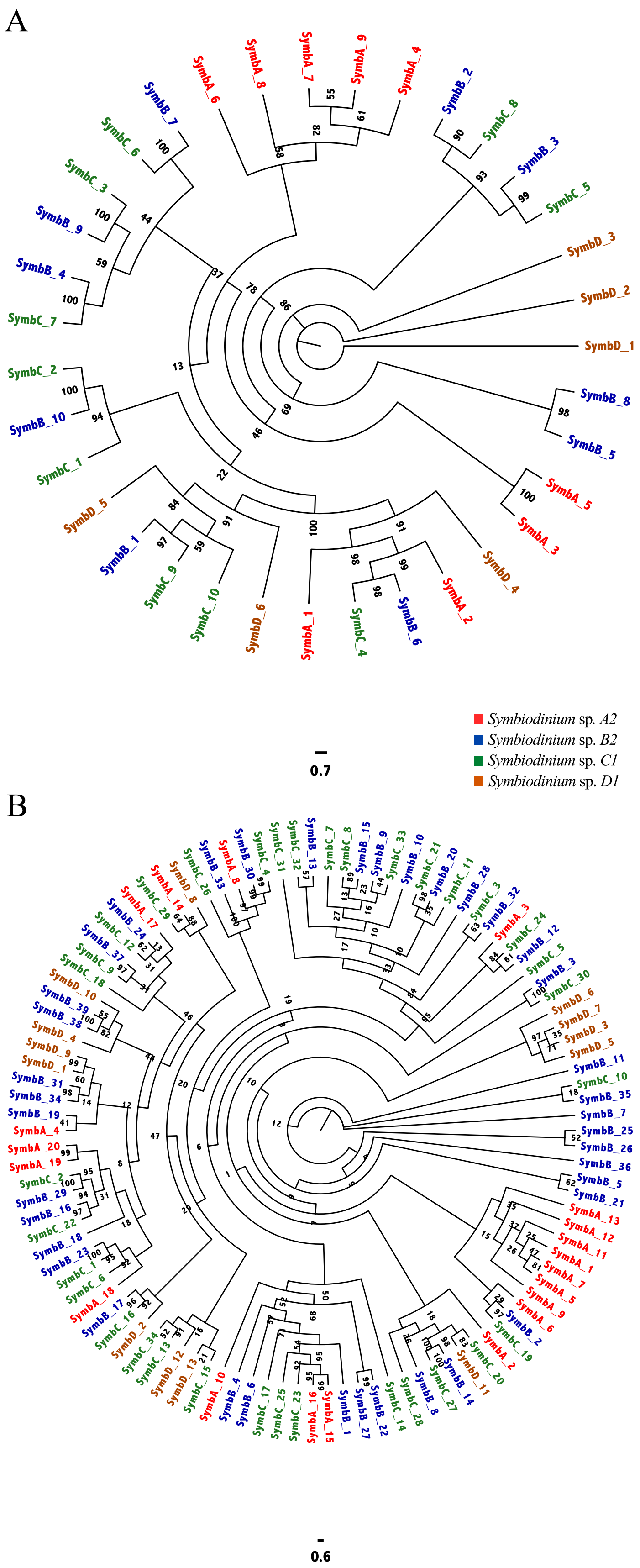
A
Symbiodinium sp. *A2*
Symbiodinium sp. *B2*
Symbiodinium sp. *C1*
Symbiodinium sp. *D1*
0.7
B
0.6

Fig. S6. Maximum-likelihood trees (1,000 bootstraps) for (A) bicarbonate transporters (PF00955 HCO3_cotransp) and (B) ammonium transporters (PF00909 Ammonium_transp) present in the transcriptomes of *Symbiodinium* spp. from Clades A, B, C, and D. Phylogenetic grouping of bicarbonate and ammonium transporters by species indicate lineage specific duplications in all *Symbiodinium* species representing different clades. Only genes and transcripts with transporter domains with e values lower than e-15 and lengths above 150 amino acids were selected for the analysis. Transcripts are colored according to species.
